# Phenotype driven data augmentation methods for transcriptomic data

**DOI:** 10.1093/bioadv/vbaf124

**Published:** 2025-05-23

**Authors:** Nikita Janakarajan, Mara Graziani, María Rodríguez Martínez

**Affiliations:** AI for Scientific Discovery, IBM Research Europe, Rüschlikon 8803, Switzerland; D-INFK, ETH Zürich, Zürich 8092, Switzerland; AI for Scientific Discovery, IBM Research Europe, Rüschlikon 8803, Switzerland; AI for Scientific Discovery, IBM Research Europe, Rüschlikon 8803, Switzerland

## Abstract

**Summary:**

The application of machine learning methods to biomedical applications has seen many successes. However, working with transcriptomic data on supervised learning tasks is challenging due to its high dimensionality, low patient numbers, and class imbalances. Machine learning models tend to overfit these data and do not generalize well on out-of-distribution samples. Data augmentation strategies help alleviate this by introducing synthetic data points and acting as regularizers. However, existing approaches are either computationally intensive, require population parametric estimates, or generate insufficiently diverse samples. To address these challenges, we introduce two classes of phenotype-driven data augmentation approaches—signature-dependent and signature-independent. The signature-dependent methods assume the existence of distinct gene signatures describing some phenotype and are simple, non-parametric, and novel data augmentation methods. The signature-independent methods are a modification of the established Gamma-Poisson and Poisson sampling methods for gene expression data. As case studies, we apply our augmentation methods to transcriptomic data of colorectal and breast cancer. Through discriminative and generative experiments with external validation, we show that our methods improve patient stratification by 5−15% over other augmentation methods in their respective cases. The study additionally provides insights into the limited benefits of over-augmenting data.

**Availability and implementation:**

Code for reproducibility is available on GitHub.

## 1 Introduction

The application of machine learning in biomedicine, particularly for supervised learning tasks pertaining to cancer, is becoming increasingly popular. Technological advances have provided increasing access to transcriptomic data, catalysing an increase in research aimed at pattern discovery, phenotype classification, disease subtyping, survival analysis, and more (Swanson *et al.* 2023). However, inherent challenges in this domain persist, notably the high dimensionality of the data and the limited number of samples (Dudoit *et al.* 2002). This is particularly problematic for supervised learning tasks because the class labels are often imbalanced in such datasets (Sun *et al.* 2009). All of these challenges combined make machine learning models prone to over-fitting and reduce their ability to generalize to new datasets (Hastie *et al.* 2009), even for the same samples measured at different locations (Sugiyama and Kawanabe 2012).

Data augmentation methods are typically used to alleviate challenges associated with limited sample sizes. These methods generate new data points from existing ones, addressing any class imbalances and under-representations. The most commonly used method is random oversampling or sampling with replacement (Efron and Tibshirani 1994). While effective for constructing accurate, less biased classifiers (Batista *et al.* 2004), this method, however, does not generate new samples, and so the model gains no new information about the underlying distribution. This affects model generalization and robustness to unseen datasets. Although new sample generation can be achieved by a weighted mixing of observations (Kircher *et al.* 2022), assigning unique class labels to such samples can be challenging, making it less suitable for supervised learning. Oversampling by interpolation is another popular method of augmenting biological data. Synthetic Minority Oversampling Technique (SMOTE) (Chawla *et al.* 2002) is a prime example of this sampling strategy, achieving state-of-the-art performances on tasks affected by class imbalances. However, the method comes with its challenges: (i) for high-dimensional datasets, SMOTE does not attenuate the bias towards the majority class (Blagus and Lusa 2013), (ii) when applied to high-dimensional data, SMOTE preserves the expected value of the minority class while decreasing its biological variability, which can be problematic for classifiers that rely on class-specific variances (Blagus and Lusa 2013), and (iii) SMOTE can be computationally demanding due to extensive pairwise distance calculations.

Data can also be augmented using methods that rely on population statistics and data models. In the case of transcriptomic data, this translates into using parametric methods such as the Poisson distribution (Marioni *et al.* 2008, Bullard *et al.* 2010, Wang *et al.* 2010) or the negative binomial distribution, also known as the Gamma-Poisson distribution (Robinson *et al.* 2010, Love *et al.* 2014) to sample new data. To solve class imbalance, these methods require class-specific parameter estimations. Challenges arise when the data distribution support is too large, leading to low data density in some regions and potentially generating uninformative data distributions. Conversely, limited support can result in insufficient sample diversity. Furthermore, out-of-distribution data points can also impact parameter inference, making these methods sensitive to outliers and measurement errors (Hendrycks and Gimpel 2017). Deep learning-based methods, such as variants of Generative Adversarial Networks (GAN) (Goodfellow *et al.* 2020), instead learn the distribution parameters to create synthetic gene expression samples (Chaudhari *et al.* 2020, Kircher *et al.* 2022, Viñas *et al.* 2022, Li *et al.* 2023). However, these methods are computationally intensive, difficult to train, and require both detailed knowledge about gene regulatory networks and a large amount of data. Such large datasets are difficult to obtain, particularly in cancer studies.

Given the challenges faced by current approaches in generating new samples for supervised learning, we propose two types of phenotype-driven approaches for data augmentation. The first type leverages phenotypic gene signatures—sets of genes with distinct expression patterns that hold prognostic, diagnostic or predictive value—to drive the augmentation. Gene signatures are invaluable for phenotypic predictions and are especially useful in cancer subtyping (Yersal and Barutca 2014, Rodriguez-Salas *et al.* 2017, Zhou *et al.* 2021). In a nutshell, patients’ gene signatures within and between phenotype variants are mixed to generate new observations. This set of methods is computationally efficient and does not require estimating distribution parameters. The second type is a modification of the parametric methods—Gamma-Poisson and Poisson sampling—for cases where signatures are not known or entirely distinct for a phenotype. To address challenges with class-imbalance, we adapt these methods such that a new distribution is initialized based on a subset of data to generate a new sample.

We demonstrate the effectiveness of our sampling methods on cancer-related tasks. We believe limitations in data availability are a contributor to its status as one of the least understood diseases. Moreover, as significant work has been done in the field to identify associated signatures, it presents us with a playground for testing signature-dependent and -independent methods, particularly in subtype identification. We make use of colorectal [TCGA COADREAD (Colon Adenocarcinoma and Rectal Adenocarcinoma)] (Network CGA 2012) and breast cancer [GSE20713 (Dedeurwaerder *et al.* 2011)] gene expression datasets for training and evaluate the generalization performance on the out-of-domain CPTAC COAD (Colon Adenocarcinoma) (Edwards *et al.* 2015), EPICC (Heide *et al.* 2022, Househam *et al.* 2022), and METABRIC (Curtis *et al.* 2012, Pereira *et al.* 2016) datasets, respectively. Our methods successfully address class imbalances, improve model performance, and offer a promising solution for data augmentation in the biomedical domain. We envision our methods to be applicable in modelling mixed phenotype scenarios, particularly useful for cancer heterogeneity studies, and in developing models that are robust against class imbalance, a problem that plagues the biology domain.

Our contributions are as follows:

1. We introduce a novel, non-parametric signature-dependent data augmentation method and benchmark it against conventional techniques;
2. We demonstrate the utility of the Gamma-Poisson and Poisson distributions as class-balancing signature-independent data augmentation strategies by proposing a modification in the sampling strategy;
3. We highlight the influence of augmentation size on model performance through a series of experiments in both discriminative and generative settings.

## 2 Methods

### 2.1 Signature-dependent sampling

Signature-dependent sampling aims to create new data points from existing data points while increasing diversity in the generated samples. Simply put, to generate new samples for a given phenotype, we leverage the high informativeness of the gene signatures associated with that particular phenotype, while assuming that the signatures of the other phenotypes in that sample are less informative for the given phenotype. We investigate two ways of augmenting the data samples:

1. *Intra-class crossover:* Crossing over between samples having the same phenotypic variant.
2. *Inter-class crossover:* Crossing over between samples across all phenotypic variants.

In the following sections, we describe the methods through a practical example of the gene expression of patients with colorectal cancer, using the Consensus Molecular Subtype (CMS) as phenotype. This phenotype has four variants, namely, CMS1, CMS2, CMS3, and CMS4 (Guinney *et al.* 2015), and each CMS class has a set of 10 genes that are highly predictive of it (Buechler *et al.* 2020). This greatly reduces the dimensionality of our gene expression dataset and gives us a panel of 40 genes that can be used for classifying patients into the four colorectal cancer subtypes. We describe the two methods of crossing over using this dataset as our premise.

#### 2.1.1 Intra-class crossover sampling

To limit the likelihood of mixing phenotypes, and hence introducing samples of dubious labels, we first perform crossing over between samples belonging to the same phenotype. Thus, in this setting, new samples of a given phenotype are generated only from samples having the same phenotype. Naturally, the synthetic sample inherits that phenotype. The crossing-over between samples is done at the gene signature level.

**Figure 1.**
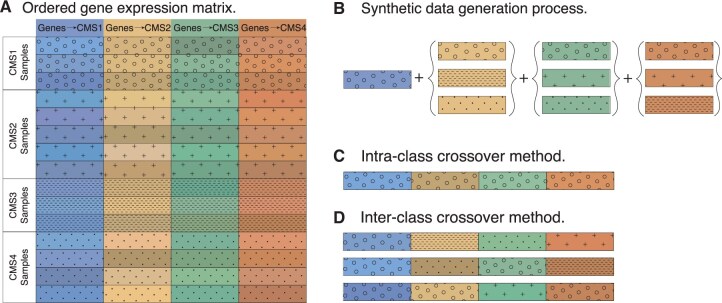
Crossover sampling strategy. (A) Consider a gene expression matrix where each gene block, identified by colour, is the predictive signature of a phenotype (colorectal cancer subtype CMS), indicated by pattern. The rows represent patients of a given phenotype, and the shade of the gene block indicates a patient-dependent expression pattern of that block. (B) We mix and match gene signature blocks to generate a new sample under certain phenotypic constraints. (C) Intra-class crossover sampling. A sample of class CMS1 is generated by sampling gene signature blocks from only the subset of CMS1 patients. (D) Inter-class crossover sampling. A sample of class CMS1 is generated by sampling the CMS1 gene signature block from only the subset of CMS1 patients, and each of the remaining gene blocks is sampled from patients who do not belong to the CMS class predicted by the gene block.

Figure 1C illustrates the process of intra-class crossover sampling. Consider an ordered gene matrix where the patients and the genes are grouped according to their CMS class. To generate a new CMS1 sample, we randomly sample each of the four gene signature blocks from only the subset of all CMS1 patients.

Since we sample the blocks only from the subset of CMS1 patients, the expression values of genes predictive of the other CMS types are typical of what is observed in a CMS1 patient. This process is repeated for the other CMS types, where a CMS2 patient is sampled only from the subset of patients belonging to CMS2, and so on. Restricting the sampling subset also allows this method to handle any correlations between genes in a consistent way. Section 1, available as supplementary data at *Bioinformatics Advances* online presents a more formal definition of the method.

#### 2.1.2 Inter-class crossover sampling

In inter-class crossover sampling, signature blocks are sampled from the complete dataset across all phenotypes. To avoid ambiguous labels, we add a constraint such that the predictive signature block can only come from a sample that belongs to the target phenotype being generated. This method utilizes the entire dataset to create new samples, in contrast to intra-class crossover sampling, which samples from a phenotype-specific subset of the data. A detailed description of the method can be found in Section 2, available as supplementary data at *Bioinformatics Advances* online.

Inter-class crossover sampling is illustrated in Fig. 1D in the context of CMS classes. When creating a CMS1 sample, we first sample the predictive signature block from the set of all CMS1 patients. This ensures that the predictive signature block exhibits expression patterns characteristic of CMS1 patients. The remaining gene blocks are sampled from all patients, except those belonging to the CMS class associated with each respective gene block. This prevents any ambiguity in class label assignments. For example, the CMS2 block can be sampled from all patients except those classified as CMS2, since the expression values of the CMS2 block in non-CMS2 patients are not typically predictive of CMS2.

### 2.2 Signature-independent sampling

Parametric methods such as the Gamma-Poisson and Poisson distributions are popular augmentation methods for RNA-Seq data. In our experiments, we modify these two methods to improve the class-specific diversity of the generated samples compared to the original versions. Briefly, for each new sample generation, the distributional parameters are estimated from randomly sampled sub-populations of the same phenotype class, enhancing variation while maintaining class characteristics.

#### 2.2.1 Modified Gamma-Poisson sampling

The negative binomial (NB) distribution is a commonly used parametric method for augmenting RNA-Seq data, typically expressed as a Gamma-Poisson mixture (Greenwood and Yule 1920, Barry 2020) to improve tractability. In this formulation, the Poisson distribution’s rate parameter λ is a random variable sampled from a gamma distribution parametrized by the shape (α) and rate (β) parameters, which are estimated from the observed population.

Our proposed modification creates a mixture of Gamma-Poisson distributions. We randomly select a subset *S* of samples belonging to the same class for each new observation. The mean μ and variance $\sigma^{2}$ of this subset *S* are used to estimate the α and β parameters, which initialize the gamma distribution as shown in Equation 1. A random variable is then sampled from this gamma distribution to serve as the rate parameter for the Poisson distribution, from which a new observation is drawn. Since the subset contains samples of the same class, the newly generated observation inherits that class label. The subset size |S|is a user-defined hyperparameter. This process is repeated *n* times to generate *n* new observations. Section 3, available as supplementary data at *Bioinformatics Advances* online provides a more detailed explanation.


(1) $$\begin{array}{c} \mu =\frac{1}{\left|S\right|}\sum_{x\in S}x,\;\sigma^{2}=\frac{1}{|S|-1}\sum_{x\in S}{\left(x-\mu \right)}^{2}\\ \alpha =\frac{\mu^{2}}{\sigma^{2}},\;\beta =\frac{\mu }{\sigma^{2}} \end{array}$$

#### 2.2.2 Modified Poisson sampling

Similar to the Gamma-Poisson strategy, a subset is first sampled from the class to be augmented, after which the mean is computed and defined as the rate parameter λ for the Poisson distribution as shown in Equation 2. A new observation is sampled from this distribution; this process is repeated *n* times to generate *n* new observations. A more detailed explanation is described in Section 4, available as supplementary data at *Bioinformatics Advances* online.


(2) $$\lambda =\frac{1}{\left|S\right|}\sum_{x\in S}x\;,\;\;Y_{j}∼\mathrm{Poisson}(\lambda_{j})$$

## 3 Experimental setting

### 3.1 Datasets

We use TCGA COADREAD (Network CGA 2012), CPTAC (Edwards *et al.* 2015), and EPICC (Heide *et al.* 2022, Househam *et al.* 2022) RNA-Seq datasets for the first part of our study, demonstrating the signature-dependent methods. TCGA COADREAD is designated for training and in-domain testing, while CPTAC COAD and EPICC gene expression datasets are used exclusively for out-of-domain validation. As an initial pre-processing step, we filter out replicates and only consider patients with primary tumours and available phenotype labels. The genes are reduced to the signature genes associated with these phenotypes. For the second part of our study addressing edge cases such as unavailable, overlapping, or correlated signatures, we utilize GSE20713 microarray breast cancer data (Dedeurwaerder *et al.* 2011) for training and in-domain testing, and METABRIC (Curtis *et al.* 2012, Pereira *et al.* 2016) dataset for out-of-domain validation. Summary of class counts of all datasets are shown in Tables 1 and 5, available as supplementary data at *Bioinformatics Advances* online, respectively.

**Table 1.** Average balanced accuracy scores and their standard deviation from 5 × 5 cross-validation for MSI and CIMP classification with class size 500 using only 10% real training data.^a^

Clinical variable	Sampling methods	Average balanced accuracy	
		TCGA	CPTAC
MSI	Mod. Gamma-Poisson	0.5912±0.0576	0.6348±0.099
	Inter-Class	0.6046±0.0562	0.7039±0.1198
	Intra-Class	0.6054±0.0594	**0.7086 ± 0.1087**
	Mod. Poisson	0.586±0.0584	0.6603±0.0907
	Replacement	**0.6127 ± 0.0533**	0.6884±0.1012
	SMOTE	0.5981±0.058	0.649±0.0972
	Unaugmented	0.4868±0.0346	0.5023±0.035
CIMP	Mod. Gamma-Poisson	0.4962±0.0571	—
	Inter-Class	**0.5228 ± 0.0624**	—
	Intra-Class	0.5102±0.0628	—
	Mod. Poisson	0.4974±0.0704	—
	Replacement	0.5095±0.0654	—
	SMOTE	0.5066±0.0711	—
	Unaugmented	0.3408±0.0427	—

a The best scores are highlighted in bold. For both MSI and CIMP classification, our crossover methods consistently rank in the top 2 for both in-domain (MSI and CIMP) and out-of-domain validation (MSI only). The results demonstrate that our crossover methods significantly improve prediction power over unaugmented data.

### 3.2 Data augmentation process

Data augmentation is applied only to training data. The external datasets CPTAC and METABRIC used for validation are not augmented. The following augmentation methods are benchmarked: (i) Inter-class crossover sampling, (ii) Intra-class crossover sampling, (iii) Modified Gamma-Poisson sampling (Mod. GP), (iv) Modified Poisson sampling (Mod. Poisson), (v) Synthetic Minority Oversampling Technique (SMOTE) (Lemaître *et al.* 2017), (vi) Random oversampling (Replacement), and (vii) No augmentation (Unaugmented). The train and in-domain test splits are created from the TCGA COADREAD count and GSE20713 RMA-normalized microarray training datasets.

In the case of TCGA COADREAD, we consider three class sizes for augmentation – ‘Max’, 500, and 5000. In the ‘Max’ setting, only the minority classes are oversampled to match the size of the majority class. All classes are augmented for class sizes of 500 and 5000 until the number of samples in each class reaches 500 and 5000, respectively. Table 2, available as supplementary data at *Bioinformatics Advances* online provides a summary of real and synthetic sample counts in the training data per class size. In addition to testing the generalizability of the augmentation methods, the different sizes also provide insights into the effect of size on the quality of the augmented data. For the unaugmented case, we use the log-FPKM normalized expression data of TCGA COADREAD. The augmented TCGA COADREAD data can be visualized in Section 5. Details on hyperparameter selection for Mod. GP and Mod. Poisson is described in Section 6. After augmenting TCGA COADREAD count data, we perform FPKM normalization (Fragments Per Kilobase per Million mapped fragments) and log transformation as per (Conesa *et al.* 2016), based on the training split.

Since GSE20713 microarray data are one order of magnitude smaller than TCGA COADREAD, augmentations are also one order of magnitude smaller; i.e. we augment the data to class sizes ‘Max’, 50, and 500. A summary of real and synthetic sample counts in the training data per class size is provided in Table 6, available as supplementary data at *Bioinformatics Advances* online. We use the RMA-normalized expression data for the unaugmented case as provided. Because microarray data from GSE20713 is already RMA-normalized, we do not do any further processing.

### 3.3 Models

For all discriminative tasks, we consider five commonly used classifiers to obtain the overall performance estimate, namely, Logistic Regression (LR), K-Nearest Neighbours (KNN), Support Vector Machines with RBF kernel (SVM-RBF), Explainable Boosting Machines (EBM), and Random Forests (RF) as implemented by sklearn. For the generative tasks, we use a Variational Autoencoder (VAE) (Kingma and Welling 2014), implemented using PyTorch.

### 3.4 Evaluation criteria

We report the balanced accuracy [Equation (5), available as supplementary data at *Bioinformatics Advances* online] for all classification tasks on the in-domain and out-of-domain test sets. The balanced accuracy is the average per-class recall (or sensitivity) score, as implemented by sklearn. If the support samples for each class exhibit sufficient and meaningful diversity, the test classification performance on the augmented data is expected to outperform that of the unaugmented dataset. We perform a five-fold stratified cross-validation repeated five times, resulting in 25 unique training-test splits for each augmentation method and augmentation size. Pairwise significance tests are conducted using a Wilcoxon signed-rank test adjusted to control the false discovery rate using the Benjamini-Hochberg method. Methods significantly better than unaugmented are indicated with * and methods significantly better than all other methods are indicated with ▲ in the figures. We also report the ROC-AUC scores and confusion matrices in the Supplementary, available as supplementary data at *Bioinformatics Advances* online, referred to in Section 4.

## 4 Results

In the first part of the experiments, we evaluate the generalization performance of our proposed methods in two cases: (i) colorectal cancer subtype prediction (CMS) with non-overlapping signatures and (ii) breast cancer PAM50 subtype prediction with overlapping signatures. In the second part of our experiments, we conduct an in-depth analysis of our methods using the colorectal cancer dataset. Implementation details of all experiments are described in Section 7, available as supplementary data at *Bioinformatics Advances* online.

### 4.1 Generalization performance

#### 4.1.1 Crossover sampling methods generalize better when signatures do not overlap

**Figure 2.**
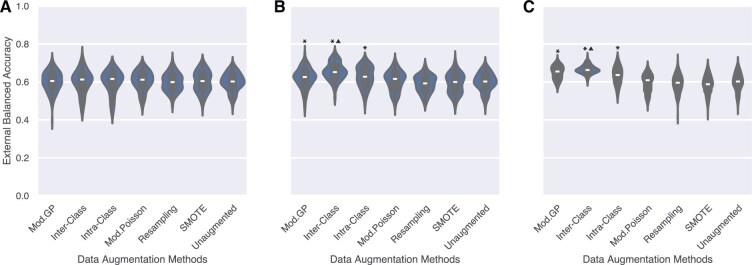
Distribution of balanced accuracy on out-of-domain CPTAC test set across all classification models (EBM, KNN, Logistic, RF, and SVM-RBF) for each augmentation method and cross-validation split for class size (A) Max, (B) 500, and (C) 5000. The trend indicates that the inter-class crossover method achieves the best generalization performance and is a good candidate for tasks needing generalization, as it can achieve the best accuracy with fewer augmented samples compared to Mod. GP. * indicates that the method is significantly better than the unaugmented data. ▲ indicates that the method is significantly better than all other methods.

We use colorectal cancer subtype prediction (CMS) to demonstrate the utility of our augmentation methods in the non-overlapping signature setting. We compare the generalization performance of the different augmentation methods in terms of balanced accuracy across the three class sizes – ‘Max’, 500, and 5000. The distribution of balanced accuracy on the unseen, out-of-domain CPTAC dataset across all classifiers and splits of data is illustrated in Fig. 2. The inter-class crossover method generalizes significantly better than the other augmentation methods. Particularly, inter-class crossover reports the highest overall performance for class sizes 500 (65.34%±0.032) and 5000 (66.45%±0.019), resulting in a 5%−6% increased accuracy over unaugmented data (average balanced accuracy 59.9%±0.0297). The method also shows a 5%−8% increased accuracy over data augmented by SMOTE (59.28%±0.0328 for class size 500, and 58.51%±0.0349 for class size 5000) and random oversampling (58.93%±0.0313 for class size 500, and 59.18%±0.0358 for class size 5000). Our results demonstrate that classifiers trained on data augmented by our methods maintain accurate classification of CMS subtypes. These augmentation approaches successfully increase sample diversity while preserving the essential phenotype-specific signals. This makes our methods particularly valuable for scenarios requiring data-driven classification, such as enhancing performance on various downstream tasks.

We also observe that over-augmenting the data to class size 5000 brings little benefit, as only the variance across the splits is reduced. However, the difference in average performance is minimal compared to class size 500, suggesting that over-augmentation offers little to no benefits at the expense of increased computation cost.

Section 8.3.1, available as supplementary data at *Bioinformatics Advances* online provides further insights into the findings. The average balanced accuracy of each classification model, augmentation method, and class size is quantified in Tables 13 and 14, available as supplementary data at *Bioinformatics Advances* online for in-domain and out-of-domain test sets, respectively. We also illustrate the ROC-AUC scores with violin plots for both in-domain (Fig. 7, available as supplementary data at *Bioinformatics Advances* online) and out-of-domain (Fig. 8, available as supplementary data at *Bioinformatics Advances* online) test sets. The confusion matrices are reported in Fig. 6, available as supplementary data at *Bioinformatics Advances* online. We evaluate all methods on a second external dataset, EPICC (Heide *et al.* 2022, Househam *et al.* 2022). The results support our findings and are discussed in Section 8.3.1, available as supplementary data at *Bioinformatics Advances* online.

#### 4.1.2 Modified Gamma-Poisson sampling is the best choice for overlapping signatures

Although having non-overlapping signatures is ideal, it is often not the case. Signature-dependent methods cannot inherently handle overlapping genes as they rely on mixing blocks of genes. Overlapping genes complicates this procedure as the class being sampled and the relationships between these genes and classes need to be taken into account. The signature-independent methods, however, face no such challenge. Since sub-populations of the same class are sampled to initialize the sampling distributions, the inherent relationships between genes are maintained.

To demonstrate the limitations and strengths of these two approaches, we define a classification problem based on PAM50 breast cancer subtypes—Luminal A, Luminal B, and Basal, where Luminal A and Luminal B signatures have two overlapping genes between them. These genes are up-regulated in the Luminal B subtype and down-regulated in Luminal A. Since the signature-dependent crossover methods cannot inherently handle overlapping genes, we use an adaptation of these methods. To generate a new sample of type {Luminal A, Luminal B} using the crossover methods, only the label-predictive signature includes the overlapping genes. To generate a Basal type that has no overlapping genes, one of the non-predictive signatures is chosen at random to contribute to the overlapping genes and maintain consistency in the expression pattern of the genes.

**Figure 3.**
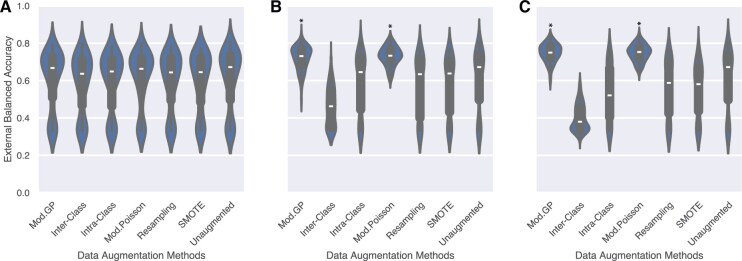
Distribution of balanced accuracy in the case of overlapping gene signatures on out-of-domain METABRIC dataset across all classification models (EBM, KNN, Logistic, RF, and SVM-RBF) for every augmentation method and cross-validation split for class size (A) Max, (B) 50, and (C) 500. The trend indicates that both Mod. GP and Poisson methods achieve the best generalization performance and are the best methods when signatures overlap. * indicates that the methods are significantly better than unaugmented data. We notice a bimodal distribution as SVM-RBF performs poorly, classifying samples at random (Supplementary Table 17).

Figure 3 illustrates the distribution of balanced accuracy scores in the cross-validation test splits on the external METABRIC dataset. The Mod. GP and Poisson methods show the best generalization performance as the augmentation size increases. These methods are significantly better than all the other methods (except each other) and show an improvement of 8−9% over models trained with unaugmented data in classifying the subtypes. The adaptation of the crossover methods for handling overlapping genes does not work, as evidenced by the poor generalization performance. We hypothesize that the correlation between genes in the signatures of Luminal A and Luminal B prevents the crossover sampling methods from maintaining fidelity in the data. Detailed results on METABRIC are described in Table 17, available as supplementary data at *Bioinformatics Advances* online. Results on in-domain testing of the augmentation methods, including the original implementations of Gamma-Poisson and Poisson sampling, are illustrated in Fig. 12, available as supplementary data at *Bioinformatics Advances* online and Table 16, available as supplementary data at *Bioinformatics Advances* online, and lead to a similar conclusion. The distribution of the ROC-AUC scores across all splits is shown in Fig. 13, available as supplementary data at *Bioinformatics Advances* online for in-domain and Fig. 14, available as supplementary data at *Bioinformatics Advances* online for out-of-domain test sets. The confusion matrices are reported in Fig. 15, available as supplementary data at *Bioinformatics Advances* online. Section 8.3.2, available as supplementary data at *Bioinformatics Advances* online provides further insights into these results.

To summarize, the inter-class crossover method should be preferred when gene signatures do not overlap, as evidenced by its significantly higher generalization power (Fig. 2). Should the gene signatures overlap, the Mod. GP method is the better choice; its ability to capture over-dispersion in the data over Poisson-based sampling (Robinson *et al.* 2010, Love *et al.* 2014) makes it preferable.

### 4.2 Evaluating synthetic data quality

First, using the augmented colorectal cancer datasets, we assess the quality of the generated synthetic data. Inspired by Zhang et al. (2024), we utilize metrics such as Shape, Trend, Detection, and Coverage as implemented by Synthetic Data Metrics (2025). The results of this analysis are shown in Table 11, available as supplementary data at *Bioinformatics Advances* online. We find that the inter-class and intra-class methods rank highly in Shape and Detection, while the Mod. GP has a perfect Coverage score and a better Trend score than inter-class sampling.

This evaluation is further supplemented by a cosine-metric based silhouette score analysis wherein we compute the difference in the silhouette scores between the real and synthetic data. The scores (Table 12, available as supplementary data at *Bioinformatics Advances* online) indicate that the intra-class, inter-class, and Mod. GP sampling methods have a lower error rate compared to SMOTE. A detailed description of the metrics and analysis discussion is provided in Section 8.2, available as supplementary data at *Bioinformatics Advances* online.

### 4.3 Effect of real data size

Next, we evaluate the impact of the initial dataset size on the different augmentation strategies. We select increasing percentages of the real training data, namely 10, 20, 30, 40, and 50%, in a stratified manner to reflect the class imbalances present in the original dataset. This simulates the ‘very low data regime’ scenario. We repeated the classification experiment for each percentage of real data and tested the models on the in-domain TCGA COADREAD test set and the unseen out-of-domain CPTAC COAD dataset at different augmented class sizes.

**Figure 4.**
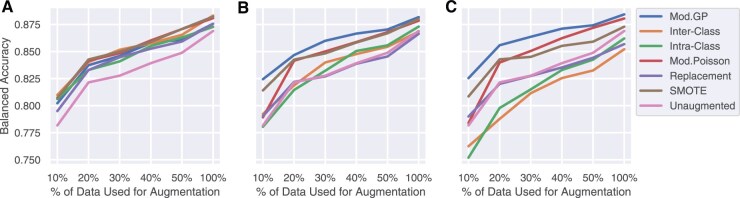
Trends in average classification performance for class size (A) Max, (B) 500, and (C) 5000 on in-domain TCGA test set as the percentage of available real training data increases. Mod. GP sampling is resistant to over-augmentation.

Figure 4 shows the in-domain averaged balanced accuracy across all classification models considered for the three class sizes at different percentages of real data in training. The trends in all three subplots are consistent with the fact that as we increase the number of real data observations, the performance of the classification models improves. Again, we see that over-augmenting the data only results in limited improvement for distribution-based methods. For instance, for the Mod. GP method on 10% of the real training data, the associated average balanced accuracy increases from 82.444%±0.0528 to 82.54%±0.0571 when augmenting from class size 500 to 5000. Detailed results of individual classification models with each augmentation method and class size for the 10% real data scenario are shown in Tables 18 and 19, available as supplementary data at *Bioinformatics Advances* online.

**Figure 5.**
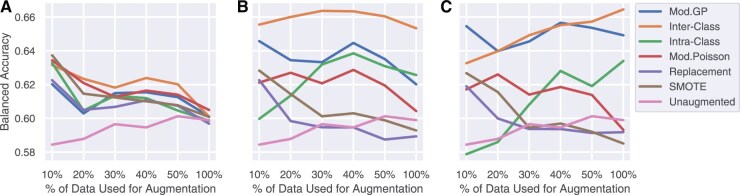
Trends in average classification performance for class size (A) Max, (B) 500, and (C) 5000 on out-of-domain CPTAC dataset as the percentage of available real training data increases. Inter-class crossover sampling is resistant to over-augmentation and produces the best performance with fewer augmented data points compared to other methods.

Figure 5 shows the out-of-domain averaged balanced accuracy across all classification models. At class sizes of 500 and 5000, the inter-class crossover method achieves the best performance. At a class size of 500, this method is achieved 65.55%±0.0492 when only 10% of the real training data is used, which is as high as that of using 100% of the data. The second-best method is Mod. GP, which achieves a similarly high performance but at class size 5000 (65.46%±0.0515).

In conclusion, the inter-class crossover method is the best for generalization, and over-augmentation does not significantly help performance other than adding to computational runtime. For some augmentation methods, it can even be detrimental to performance. We find that augmenting each class to size 500 proves to be optimal for all augmentation methods. Henceforth, we carry out further experiments using this class size.

### 4.4 Mixing gene blocks has added value

In this experiment, we analyse whether the crossover sampling strategy interferes with the biological signal of the sample. We consider the scenario where only 10% of the real training data (≈40 samples) is available. Since we retain the gene blocks as is, we do not destroy phenotype-specific information through mixing. We demonstrate this point by showing that key clinical variables of colorectal cancer subtypes, namely, microsatellite instability (MSI) and CpG Island Methylator Phenotype (CIMP), are still predictable. Predicting these variables, among others such as demographic factors, genomic markers, and treatment response, is relevant for patient prognostics, disease monitoring, and disease subtyping (Popat *et al.* 2005, Min *et al.* 2011, Shiovitz *et al.* 2014, Kang *et al.* 2018, Sillo *et al.* 2019, Diao *et al.* 2021). Since we cannot infer MSI and CIMP labels for augmented data points, we first train a VAE using the augmented data (40-dimensional), retrieve the compressed embeddings (4-dimensional) of real data points, and train classifiers on these embeddings. Thus, we indirectly evaluate the ability of augmentation methods to preserve information from the subtypes.

Aggregated results are shown in Table 1, where we report the average balanced accuracy across all classifiers in predicting the MSI and CIMP status of patients. The inter-class and intra-class augmentation methods show a significant improvement over unaugmented data - 12% in-domain and 20% out-of-domain for MSI prediction, and 18% in-domain for CIMP prediction. These results prove that the mixing of gene blocks between samples does not drastically affect the predictiveness of other variables associated with these colorectal cancer subtypes. Results for each classifier and augmentation method can be found in Tables 22 and 23, available as supplementary data at *Bioinformatics Advances* online for in-domain and out-of-domain MSI prediction, respectively, and Table 24, available as supplementary data at *Bioinformatics Advances* online for in-domain CIMP prediction.

### 4.5 Generation quality

Finally, we evaluate the augmentation methods in the generative modelling task where only 10% of real training data are available. Here, we assess how effectively the augmentation contributes to estimating a parametric representation of the data. Our hypothesis is that greater diversity in the input samples will lead to more accurate parametric estimates of the underlying distribution. To verify this hypothesis, we extract test data embeddings from a pre-trained VAE, setting the class size to 500 and using 10% of the real training data. We compute the mean and standard deviation of these embeddings grouped by their true CMS class and initialize a Gaussian-Mixture model. From this model, we sample embeddings, which are then decoded to generate new data. This procedure is performed for both TCGA and CPTAC test data. These newly generated data are then passed through our previously trained classifiers from Section 4.1.

**Figure 6.**
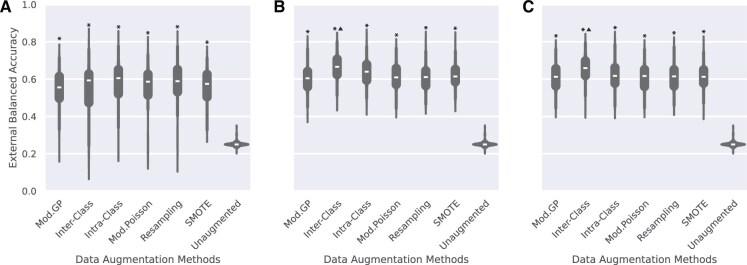
Classification performance for class size (A) Max, (B) 500, and (C) 5000 on generated samples from a VAE pre-trained on augmented data, sampled from a distribution modelled on out-of-domain CPTAC test set. On average, the inter-class crossover sampling is associated with the best performance (66.36%±0.0631). * indicates that the method is significantly better than unaugmented data. ▲ indicates that the method is significantly better than all other methods.

A considerable performance difference is seen on the out-of-domain CPTAC dataset as shown in Fig. 6. The inter-class crossover method (average balanced accuracy 66.36%±0.0631) performs the best over other methods, with an increase in accuracy of ≈35% over unaugmented data, ≈6% over Mod. GP, Mod. Poisson and replacement methods, and ≈5% over SMOTE. The results of the experiment are shown in Tables 20 and 21, available as supplementary data at *Bioinformatics Advances* online. A high classification accuracy indicates that the VAE was trained on data of sufficient quality to capture the underlying distribution. Figure 16, available as supplementary data at *Bioinformatics Advances* online shows the average balanced accuracy achieved by each model on generated samples modelled on the in-domain TCGA test set.

### 4.6 Computational complexity

In this section, we analyse the computational complexity of the augmentation methods. As the crossover methods sample signature blocks from a uniform distribution, both methods have a runtime complexity of O(pk) where *p* is the number of phenotypes and *k* is the number of new samples to be generated. Thus, the complexity is linear in both k (for fixed p) and p (for fixed k). The parametric methods have a runtime complexity of O(psnk)+O(k), where *p* is the number of phenotypes, s=|S|, the number of genes in the signature, and *n* is the number of samples from which distribution parameters are computed. The first term is the complexity of computing distribution parameters, and the second term is the complexity of sampling values from the distribution. The runtime complexity is linear since *s*, *p*, *n*, and *k* are independent. The random oversampling method has a runtime complexity of O(kn) since we generate *k* new samples and for each sample, we go through the *n* values in the dataset for sampling. SMOTE has a quadratic runtime complexity since it requires the computation of a pairwise distance matrix, and is dependent on the number of samples, n, in the dataset $\left(O(n^{2})\right)$. Empirical runtimes are discussed in Section 7.4, available as supplementary data at *Bioinformatics Advances* online and shown in Table 7, available as supplementary data at *Bioinformatics Advances* online.

## 5 Discussion

We introduce two classes of data augmentation strategies—signature-dependent and signature-independent methods. The goal of our augmentation approaches is to show how any classifier can achieve state-of-the-art performance when trained on augmented data, notwithstanding the limitations of real data. When distinct, non-overlapping gene signatures exist for target phenotypes, our signature-dependent sampling methods generate synthetic data that allows models to significantly generalize well to unseen, out-of-domain datasets. By extending our experiments to other clinical variables such as MSI and CIMP, we further demonstrate the benefits of our augmentation methods in preserving the underlying characteristics of the data. In the event that such signatures overlap, correlate, or are unavailable entirely, our signature-independent Mod. GP sampling method is the ideal choice, which significantly outperforms other commonly used data augmentation methods in classifying phenotypes with overlapping gene signatures. Although the Mod. Poisson sampling method sometimes performs on par, particularly in the lower augmentation size regime, Mod. GP should be the preferred method as it can handle over-dispersion in the data. Our experimental results highlight our augmentation methods as cost-effective alternatives to collecting additional real data points, which is considerably more resource-intensive. During the course of our experiments, we also found that over-augmenting data beyond 1–2 orders of magnitude has little to no benefit for models of limited complexity. It typically leads to <1−2% performance gain at increased computational cost, irrespective of the augmentation method. Our study shows that over-augmenting with generally well-performing methods like random oversampling and SMOTE can lead to worse generalization performance, highlighting the importance of picking the right augmentation method on a case-by-case basis.

### 5.1 Limitations

The reliance of the signature-dependent methods on known, non-overlapping gene signatures is a limiting factor to their broad use. Their inability to take into account inter-dependencies between genes from other signatures makes the method fail when applied naively to tasks where signatures have overlapping genes. Their effectiveness may be further constrained by closely related phenotypes and correlations between signature genes. The relatively poor performance on breast cancer microarray data suggests that applying these methods to RMA-normalized data violates assumptions of the normalization method, which depends on both the probes analysed and the sample count. Meanwhile, the signature-independent Mod. GP and Mod. Poisson methods rely on class-conditioned data subsets to generate new samples. We suspect that if a class is underrepresented in the dataset, these parametric methods would likely revert to their unmodified versions. Thus, the subset size is a hyperparameter that must be chosen carefully.

### 5.2 Future scope

A deeper analysis into adaptations of the signature-dependent methods to handle overlapping and correlating genes could help make the methods more versatile. Another interesting study relates to the effect of applying various expression-normalization methods before or after augmentation using the different augmentation strategies. Further research on extending these methods to the entire genome without phenotype conditioning would provide additional insights into their applicability to unsupervised learning. Our proposed sampling methods are intuitive, simple, and computationally efficient, outperforming state-of-the-art approaches and showing strong potential for applications in heterogeneity studies and disease analysis. Creating a landscape of mixed phenotypes would present new opportunities in unsupervised and semi-supervised learning in scarce data regimes, given fully unlabelled data.

## Supplementary Material

vbaf124_Supplementary_Data

## Data Availability

The code is available on GitHub (https://github.com/PaccMann/transcriptomic_signature_sampling) for reproducibility. Data and results of experiments are available on Zenodo (https://zenodo.org/records/14983178).
