# Supplementary material for: Phenotype driven data augmentation methods for transcriptomic data
Source: Bioinform Adv. 2025 May 23;5(1):vbaf124. doi: 10.1093/bioadv/vbaf124 (PMC12141816; doi:10.1093/bioadv/vbaf124)
Supplement: vbaf124_Supplementary_Data [file vbaf124_supplementary_data.zip › PDDAT_Supplementary_Material.pdf]

Supplementary Material for Phenotype Driven Data Augmentation Methods for Transcriptomic Data

Nikita Janakarajan, Mara Graziani, María Rodríguez Martínez

1 Intra-class crossover sampling formalisation

Consider a dataset with n samples x_i, where $i \in 1, ..., n$, and m phenotype variants P_j, where $j \in 1, ..., m$. Each sample x_i is of one phenotype P_j. Given each variant P_j is associated with a gene signature S_j, we represent each sample by the set of signatures associated with all possible variants, that is, $x_i = \{S_1, ..., S_m\}$. The number of genes in a signature is given by $|S_j|$. Therefore, each patient is represented by $\sum_{j=1}^{m} |S_j|$ genes. For the sake of simplicity, the genes are ordered by signature - all genes belonging to signature S_1 come first and so on, thus creating signature blocks, one for each phenotypic variant. To generate a new sample, x_{n+1} with phenotype $P_{j=1}$, we first subset all samples, belonging to this phenotype (Equation 1). This subset, $X_{j=1}$, is analogous to a bag of signature blocks (Equation 2).

$$X_{j=1} = \{x_i : x_i \in P_{j=1} \forall i \in 1, ..., n\} \quad (1)$$

$$X_{j=1} = \{S_{ij} : j \in 1, ..., m \forall i : x_i \in P_{j=1}\} \quad (2)$$

We randomly sample signature blocks from this subset (Equation 3) which are put together to create a new sample x_{n+1}.

$$x_{n+1} = \{S_j \in_R X_{j=1} \forall j \in 1, ..., m\} \quad (3)$$

We illustrate a practical example on the gene expression of patients with colorectal cancer, using the Consensus Molecular Subtype (CMS) as phenotype in Section 2.1.1 of the manuscript.

2 Inter-class crossover sampling formalisation

Consider a similar premise as the one described in Section 2.1.1 of the manuscript. To generate a synthetic sample of phenotype $P_{j=1}$, we sample $S_{j=1}$ only from the subset of patients, $X_{j=1}$, that have phenotype $P_{j=1}$, that is. $S_j \in_R X_j$. We then randomly sample the remainder signatures S_k for $k \in 1, ..., m$ and $k \neq j$, from all samples except those that belong to phenotype P_k as described in Equation 4. The sampled signatures are then put together to create a new synthetic sample, $x_{n+1} = \{S_j, S_k\}$ for $k \in 1, ..., m$ and $k \neq j$ and where $j = 1$. We illustrate a practical example on the gene expression of patients with colorectal cancer, using the Consensus Molecular Subtype (CMS) as phenotype in Section 2.1.2 of the manuscript.

$$S_k \in_R \{X - X_k\} \; \forall k \in 1, ..., m \text{ and } k \neq j \quad (4)$$

3 Modified Gamma-Poisson sampling

The negative-binomial (NB) distribution is a commonly used parametric method for augmenting RNA-Seq data for differential expression analysis due to its ability to capture the overdispersion typically seen in these data. To model the RNA-Seq read frequency Y of a given gene j, the distribution relies on two parameters, namely the mean and dispersion of the counts, i.e, $Y_j = \text{NB}(\mu_j, \phi)$.

Here, ϕ is the dispersion parameter that controls the variance of the counts, $Var(Y_j) = \mu_j + \phi\mu_j^2$. The estimation of ϕ is, however, not trivial. The negative-binomial (NB) distribution is typically expressed as a Gamma-Poisson mixture [Greenwood and Yule, 1920, Barry, 2020] to make it more tractable. In this formulation, the rate parameter λ of the Poisson distribution is a random variable sampled from a gamma distribution parametrised by the shape (α) and rate (β) parameters, which are estimated from the observed population, $\lambda \sim \Gamma(\alpha, \beta)$. The RNA-Seq read counts are then sampled from this initialised Poisson distribution, $Y_j \sim Poisson(\lambda_j)$.

To ensure that the newly generated samples add some diversity while maintaining the same distribution as the augmented class, we create a mixture of Gamma-Poisson distributions. In this strategy, to generate every new observation, a subset S of samples is randomly chosen to estimate the α and β parameters from its mean μ and variance σ^2 as shown in Equation 1 in the manuscript. These parameters initialise the gamma distribution. Since the augmentation is aimed at solving class imbalance, the subset is created from samples belonging to the same class. Thus, the newly generated observation will have the same label as the subset. The size of the subset $|S|$ is a hyperparameter defined by the user. The random variable sampled from this gamma distribution is used to initialise the Poisson distribution from which a new observation is sampled. This process is repeated n times to generate n new observations. We recommend a smaller subset size to maximise the variance in the subset. A larger variance would mean more scattered observations, thereby generating a diverse set of samples as opposed to dense local clusters with a smaller variance.

4 Modified Poisson sampling

The Poisson distribution is another method commonly used to model RNA-Seq read counts. Similarly to the Gamma-Poisson strategy, a subset is first sampled from the class to be augmented, after which the mean is computed and set as the rate parameter λ for the Poisson distribution as shown in Equation 2 in the manuscript.

However, unlike the Gamma-Poisson, this distribution assumes the variance of the sample equals the mean and fails to capture overdispersion in the data. To generate new observations with this method, a larger subset size is recommended such that the variance is closer to the mean while still being high enough to generate variability in the new data.

5 Visualising augmented data for other class sizes

Figure 1 illustrates the UMAP projected distributions of the TCGA COADREAD dataset augmented by various methods to class size 5000. These visualisations are compared to the UMAP projection of the unaugmented dataset to ascertain visually if global and local trends are fairly captured. Similar visualisation are also show for class size Max in Figure 2 and class size 500 in Figure 3.

Table 1: Sample counts of the different colorectal cancer subtypes in the TCGA, CPTAC and EPICC gene expression datasets.

Datasets	CMS1	CMS2	CMS3	CMS4	Total
TCGA	74	216	74	142	506
CPTAC	14	33	16	22	85
EPICC	28	85	17	8	138

Table 2: Sample counts of real and synthetic data in TCGA COADREAD training dataset per class size, when using 100% of the data.

Class-Size	Real	Synthetic	Total	Factor Increase
Max	404	288	692	1
500	404	1596	2000	2.89
5000	404	19596	20000	28.9

6 Distribution size effect

In this experiment, we test various reference set sizes to understand its impact on CMS classification performance. The reference set sizes considered are $r = 3, 5, 10, 25, 50$. We choose $class\_size = 5000$ so that there are enough newly sampled observations to draw a conclusion about the effect of reference set size. The study is conducted on a classification task with various classifiers and the 5-fold cross-validation balanced accuracy is reported in Table 3. The reference set size with the highest accuracy on average, $r = 5$, is chosen for the Gamma-Poisson sampling method. Figure 4 illustrates what the distribution looks like with different reference set sizes. We observe that for $r = 5$, the real and synthetic samples are well interspersed with each other showing qualitatively that the distribution of synthetic samples is similar to that of the real samples. As r increases, new samples are created that seemingly fall away from its member class resulting in new sub-clusters as seen in Figure 4c and Figure 4d, for CMS1 and CMS2 classes.

For the Poisson distribution, we double the reference set size to $r = 10$ such that the variance is more stable and closer to the mean. Figure 5 illustrates the Poisson augmented data at different reference set sizes. Here, we make a similar observation that as r increases, sub-clusters begin to form due to synthetic samples being generated well outside its member class distribution. On comparing the Poisson augmented data to the Gamma-Poisson augmented data, we observe that the CMS clusters appear to be well separated with the Poisson. While this may appear to be beneficial for certain tasks that are strictly associated with the these types, it does not capture the distribution of the real data well (Figure 1A). This could introduce spurious associations and attributions.

While we acknowledge that conclusions drawn from UMAP visualisations have to be taken with a grain of salt, we note that all UMAPs were generated with the same hyperparameter choice, differing only in their input data. This gives us a qualitative insight into what these differently augmented datasets with various reference set sizes look like and whether or not the newly generated data improve the support of a given class.

We omit results for $r = 3$ due to high failure rates. Since the parametric distributions depend on the mean and variance of the subsample, it can sometimes happen that when $r = 3$ the variance associated with the subsample is too small, resulting in NaNs being sampled from the gamma

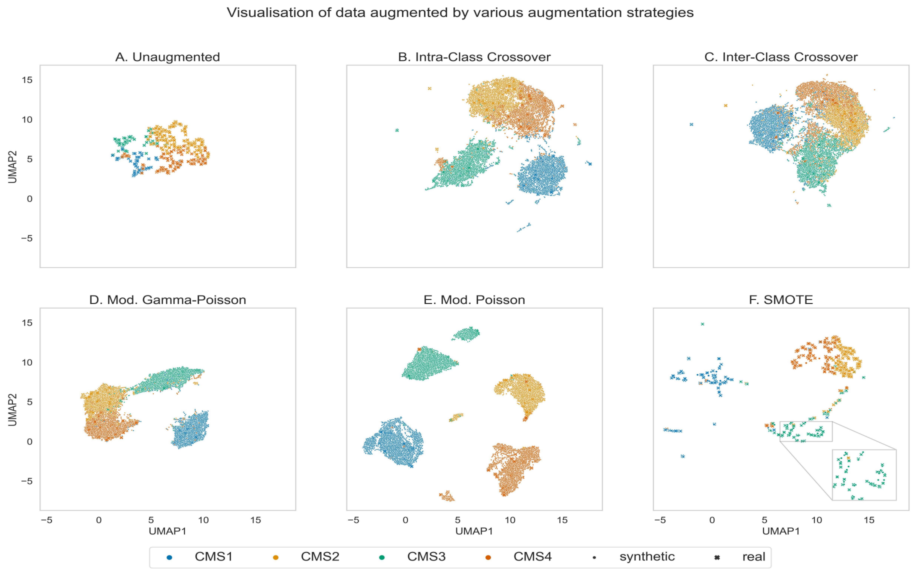

Figure 1: **UMAP [McInnes et al., 2018] visualisations of the TCGA COADREAD dataset augmented to have 5000 samples per class.** The inter-class crossover sampling method best captures both the global and local structure of the original unaugmented data.

distribution due to an invalid argument (such as 0 due to an overflow error).

Table 3: Effect of reference sample size on classifier performance for parametric methods. The table shows 5-fold cross-validation results including standard deviation for different r sizes and classifiers. Majority of the best performances belong to $\mathbf{r = 5}$, which also has the best performance on average.

Model	$r = 5$	$r = 10$	$r = 25$	$r = 50$
KNN	0.857 ± 0.035	0.836 ± 0.027	0.836 ± 0.036	$\mathbf{0.858 \pm 0.029}$
LR	$\mathbf{0.889 \pm 0.031}$	0.881 ± 0.040	0.886 ± 0.033	0.888 ± 0.049
SVM	0.886 ± 0.027	$\mathbf{0.893 \pm 0.033}$	0.889 ± 0.027	0.887 ± 0.036
RF	$\mathbf{0.881 \pm 0.029}$	0.865 ± 0.036	0.857 ± 0.037	0.859 ± 0.035
EBM	$\mathbf{0.905 \pm 0.011}$	0.892 ± 0.028	0.881 ± 0.032	0.882 ± 0.030
MLP	0.881 ± 0.020	0.889 ± 0.040	$\mathbf{0.889 \pm 0.025}$	0.883 ± 0.038

7 Implementation details

7.1 Classification performance

To demonstrate the utility of our augmentation methods in discriminative modelling, we make use of five classifiers, namely, Logistic Regression (LR), K-Nearest Neighbours (KNN), Support Vector Machines (SVM), Explainable Boosting Machines (EBM), and Random Forests (RF). For the stan-

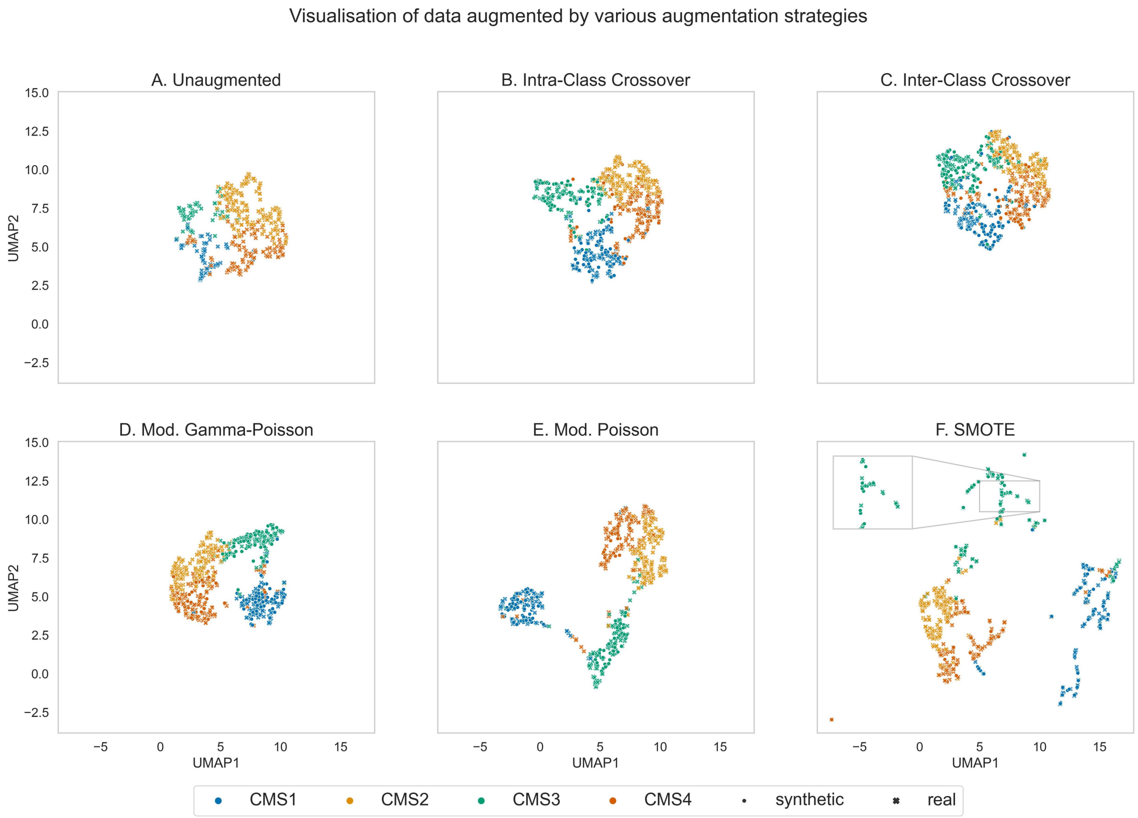

Figure 2: **UMAP [McInnes et al., 2018] visualisations of the TCGA COADREAD dataset augmented to match the majority class size.** The intra-class and inter-class crossover methods capture both the global and local trends in the original unaugmented data better than the other methods. Mod. GP and SMOTE closely follow. The Mod. Poisson sampling method begins to separate clusters which become more apparent and amplified as the class augmentation size is increased.

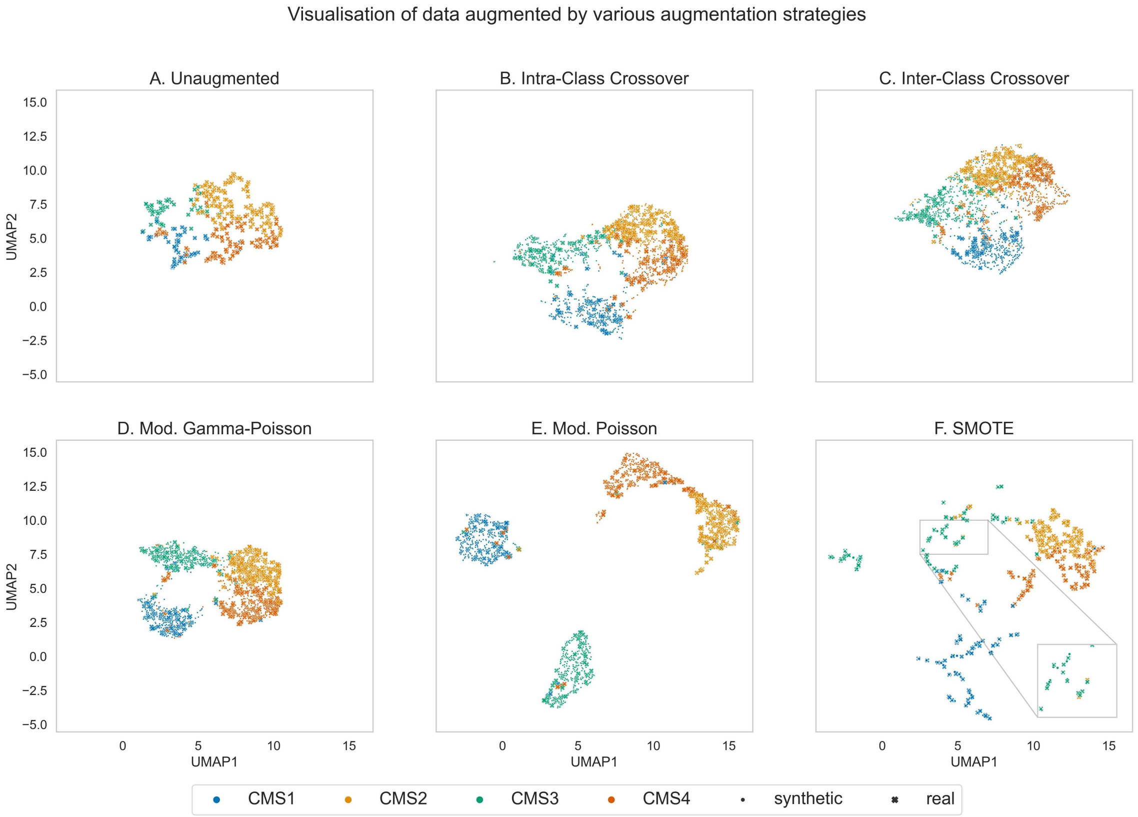

Figure 3: **UMAP [McInnes et al., 2018] visualisations of the TCGA COADREAD dataset augmented to have 500 samples per class.** Data augmented by the intra-class, inter-class crossover methods and Mod. GP sampling maintain a structure similar to the original unaugmented data. Data generated by the Mod. Poisson sampling method begins to show cluster separation, which could lead to spurious results. SMOTE generates samples on the line between two reference points and thus cannot populate sub-regions densely.

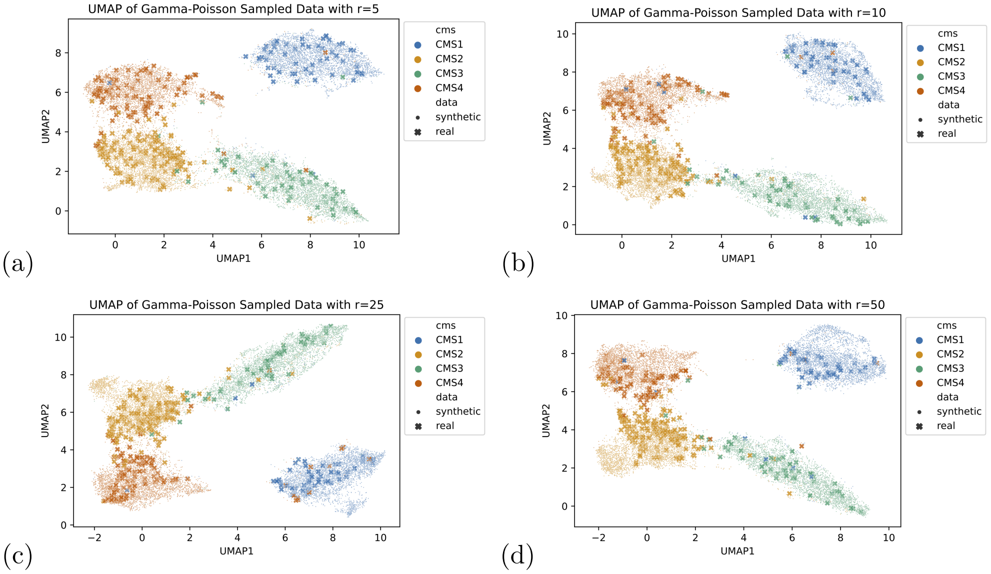

Figure 4: UMAP [McInnes et al., 2018] visualisations of Gamma-Poisson augmented TCGA COAD-READ datasets in the study. (a)r=5. (b) r=10. (c) r=25. (d) r=50. All augmented datasets have 5000 samples per class. When r is set to 5, the new samples are more interspersed with the real data, as opposed to r=50, where new samples are generated ”out of distribution” resulting in new clusters.

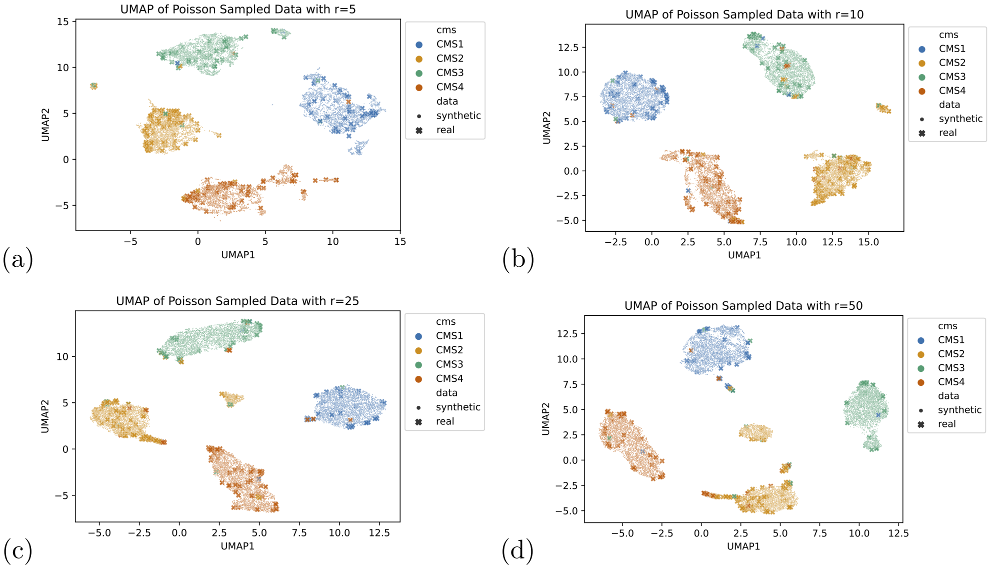

Figure 5: UMAP [McInnes et al., 2018] visualisations of Poisson augmented TCGA COADREAD datasets in the study. (a)r=5. (b) r=10. (c) r=25. (d) r=50. All augmented datasets have 5000 samples per class. For r=10, the new samples appear better interspersed with the real data and there are fewer outlier clusters.

dard classifiers, we make use of the `scikit-learn` [Pedregosa et al., 2011] library implementations with the default parameters. We only increase the `max_iter` to 5000 for the Logistic Regression model. For the EBMs, we use the `InterpretML` library. We set a seed (42) for all models for reproducibility.

We report the mean balanced accuracy (Equation 5) on the TCGA standardised test sets and the external CPTAC dataset. The metric is computed using true positive (TP), true negative (TN), false positive (FP) and false negative (FN) counts and is averaged across the 25 splits (five-fold cross-validation repeated five times). The CPTAC dataset is standardised according to the training data mean and standard deviation from each split after which it is passed to the trained model from that split. We drop patients for whom the target label is not available. We perform pairwise significance testing with the Wilcoxon signed-rank test adjusted to control the false discovery rate using the Benjamini-Hochberg method. Methods significantly better than unaugmented are indicated with $*$ and methods significantly better than all other methods are indicated with ▲ in the figures. Additionally, we compute the ROC-AUC scores and confusion matrices as implemented by the `scikit-learn` [Pedregosa et al., 2011] package. All experiments are run locally on a 2019 MacBook Pro.

$$balanced - accuracy = \frac{1}{2}\left(\frac{TP}{TP+FN} + \frac{TN}{TN+FP}\right) \quad (5)$$

Table 4: Summary counts of patients with MSI and CIMP labels available in the TCGA COAD-READ and CPTAC COAD gene expression datasets.

Clinical Variable	Categories	TCGA	CPTAC
MSI	MSI-H	80	24
	MSI-L	74	-
	MSS	339	81
	Total	493	105
CIMP	CIMP.High	66	-
	CIMP.Low	86	-
	CIMP.Neg	272	-
	Total	364	-

Table 5: Sample counts of the different breast cancer subtypes in the GSE20713 and METABRIC gene expression datasets.

Datasets	LuminalA	LuminalB	Basal	Total
GSE20713	23	22	22	67
METABRIC	700	475	209	1384

7.1.1 Effect of Data Size

The process described above is repeated for augmented data containing various percentages of real data from 10 to 50 percent and 100 percent.

7.1.2 Predicting Other Variables

For the classification tasks of predicting MSI and CIMP status, we use the SVM-RBF and EBM models from `scikit-learn` and `InterpretML` to run our experiments (with the default parameters and seed 42). Here the input to the models is a 4D latent representation extracted from the VAE (described in Section 7.3) for each patient. Similar to the survival analysis task, we perform training and testing with the same 5x5 stratified cross-validation splits, and all samples for which the MSI/CIMP status is not available are dropped for the MSI/CIMP prediction task, respectively. The patient summary counts for both MSI and CIMP are reported in Table 4.

7.2 Handling overlapping genes

The sample counts for GSE20713 and METABRIC breast cancer datasets are described in Table 5.

We use GSE20713 as the training dataset, despite its low sample number, because it presents the perfect scenario of a dataset lacking in samples, thereby warranting augmentation.

To demonstrate the utility of our augmentation methods in handling overlapping gene signatures, we design a classification experiment using the methods described in Section 3.3 of the manuscript, namely, Logistic Regression (LR), K-Nearest Neighbours (KNN), Support Vector Machines (SVM), Explainable Boosting Machines (EBM), and Random Forests (RF). For the standard classifiers, we

Table 6: Sample counts of real and synthetic data in the GSE20713 training dataset per class size.

Class-Size	Real	Synthetic	Total	Factor Increase
Max	53	1	54	1
50	53	97	150	2.7
500	53	1447	1500	27.7

make use of the `scikit-learn` [Pedregosa et al., 2011] library implementations with the default parameters. We only increase the `max_iter` to 5000 for the Logistic Regression model. For the EBMs, we use the `InterpretML` library. We set a seed (42) for all models.

We report the mean balanced accuracy (Equation 5) on the GSE20713 standardised test sets and the METABRIC microarray dataset. The metric is computed using true positive (TP), true negative (TN), false positive (FP) and false negative (FN) counts and is averaged across the 25 splits (5-fold cross-validation repeated 5 times). The METABRIC dataset is standardised according to the GSE20713 training data mean and standard deviation from each split after which it is passed to the trained model from that split. We drop patients for whom the target label is not available. Additionally, we perform pairwise significance testing with the Wilcoxon signed-rank test adjusted to control the false discovery rate using the Benjamini-Hochberg method. Methods significantly better than unaugmented are indicated with $*$. Additionally, we compute the ROC-AUC scores and confusion matrices as implemented by the `scikit-learn` [Pedregosa et al., 2011] package. All experiments are run locally on a 2019 MacBook Pro.

7.3 Generation quality

The encoder and decoder of the VAE are single-layer neural networks with 20 hidden units activated by the `ReLU` function [Agarap, 2018]. The dimension of the latent representation is 4. The batch size and learning rate are adapted according to the size of the dataset. For the unaugmented dataset and for the augmented datasets with class sizes 'Max' and '500', we set the batch size to 64 and learning rate to 0.001. For all the other datasets, we set the batch size to 128 and learning rate to 0.0001. The models are trained on the sum of the L2 loss and KL-divergence. An L2 penalty of 0.0001 is applied to all models, which are optimised by Adam [Kingma and Ba, 2014] and trained for 300 epochs with an early stopping wait time of 8 steps. The experiments to train a VAE were run on CPUs with 24 cores. The experiment for generating new samples and classifying them were run on 12 core CPUs.

7.4 Empirical runtime

In this experiment, we measured the time taken to generate an augmented colorectal cancer dataset of class size 5000 for each of the 4 CMS classes given 100% of the original data ($\approx$ 450 samples). The runtime is measured for each of the 25 cross-validation splits and averaged. We would like to note that our code implementations of the signature-dependent and signature-independent methods may not be efficient and so these methods (except intra-class crossover) appear to be slower than SMOTE. The empirical runtimes are shown in Table 7.

Table 7: Empirical runtimes of the different augmentation methods averaged over 25 splits given 100% of the data.

Sampling Method	Time (seconds)
Mod. Gamma-Poisson	136.348 ± 2.321
Inter-Class	0.642 ± 0.028
Intra-Class	0.066 ± 0.004
Mod. Poisson	60.874 ± 0.824
Replacement	0.029 ± 0.003
SMOTE	0.151 ± 0.049

Table 8: Average computational time for the crossover techniques to generate a colorectal cancer dataset of class size 5000 for each of the 4 CMS classes (repeated 3 times) when given **10%** ($\approx$ **40** samples) and **100%** of the data ($\approx$ **450** samples).

Crossover Type	Computational Time (seconds)	
	10% of Data	100% of Data
Inter-Class	0.6039	0.6202
Intra-Class	0.0707	0.0803

7.4.1 Scalability of crossover methods

To test scalability, we measured the time taken to generate an augmented colorectal cancer dataset of class size 5000 for each of the 4 CMS classes in 2 scenarios (repeated 3 times), namely, given 10% of the original data ($\approx$ 40 samples) and given 100% of the original data ($\approx$ 450 samples). Empirical results show that a 10-fold increase in dataset size results in a 2.69% increase in time taken to generate the dataset (Table 8). These results suggest that our method is scalable to larger datasets without incurring drastic computational costs. Optimising the code could achiever faster runtimes.

8 Extended results

In this section, we include long-form tables that quantify metrics for figures in the main paper as well as supporting and additional results for the experiments conducted. The aggregated metrics are reported along with their standard deviation.

8.1 Unmodified Gamma-Poisson and Poisson methods perform worse than modified

Table 9 describes the results of CMS classification of the unmodified GP and Poisson methods on in-domain TCGA test set. Table 10 describes the results of CMS classification of the unmodified GP and Poisson methods on out-of-domain CPTAC test set. Comparing these results to that of the modified versions (Table 13 and Table 14 for TCGA and CPTAC, respectively), we see how the unmodified version of Gamma-Poisson and Poisson show poorer performance compared to the modified versions.

Table 9: Average 5x5 cross-validation balanced accuracy and standard deviation on in-domain TCGA test set for the unmodified Gamma-Poisson and Poisson augmentation methods. The scores are reported for different classifier models and different class sizes. The data was augmented given 10% of the original dataset.

Class Size	Sampling Methods	EBM	KNN	Logistic	RF	SVM-RBF	Average
max	Gamma-Poisson	0.7913 ± 0.0519	0.7913 ± 0.0519	0.7913 ± 0.0519	0.7913 ± 0.0519	0.7913 ± 0.0519	0.7913 ± 0.0519
	Poisson	0.8009 ± 0.0563	0.8009 ± 0.0563	0.8009 ± 0.0563	0.8009 ± 0.0563	0.8009 ± 0.0563	0.8009 ± 0.0563
500	Gamma-Poisson	0.7982 ± 0.0460	0.7982 ± 0.0460	0.7982 ± 0.0460	0.7982 ± 0.0460	0.7982 ± 0.0460	0.7982 ± 0.0460
	Poisson	0.8196 ± 0.0615	0.8196 ± 0.0615	0.8196 ± 0.0615	0.8196 ± 0.0615	0.8196 ± 0.0615	0.8196 ± 0.0615
5000	Gamma-Poisson	0.7972 ± 0.0574	0.7972 ± 0.0574	0.7972 ± 0.0574	0.7972 ± 0.0574	0.7972 ± 0.0574	0.7972 ± 0.0574
	Poisson	0.8270 ± 0.0525	0.8270 ± 0.0525	0.8270 ± 0.0525	0.8270 ± 0.0525	0.8270 ± 0.0525	0.8270 ± 0.0525

Table 10: Average 5x5 cross-validation balanced accuracy and standard deviation on out-of-domain CPTAC test set for the unmodified Gamma-Poisson and Poisson augmentation methods. The scores are reported for different classifier models and different class sizes. The data was augmented given 10% of the original dataset.

Class Size	Sampling Methods	EBM	KNN	Logistic	RF	SVM-RBF	Average
max	Gamma-Poisson	0.6463 ± 0.0590	0.6463 ± 0.0590	0.6463 ± 0.0590	0.6463 ± 0.0590	0.6463 ± 0.0590	0.6463 ± 0.0590
	Poisson	0.6447 ± 0.0518	0.6447 ± 0.0518	0.6447 ± 0.0518	0.6447 ± 0.0518	0.6447 ± 0.0518	0.6447 ± 0.0518
500	Gamma-Poisson	0.6190 ± 0.0631	0.6190 ± 0.0631	0.6190 ± 0.0631	0.6190 ± 0.0631	0.6190 ± 0.0631	0.6190 ± 0.0631
	Poisson	0.6375 ± 0.0509	0.6375 ± 0.0509	0.6375 ± 0.0509	0.6375 ± 0.0509	0.6375 ± 0.0509	0.6375 ± 0.0509
5000	Gamma-Poisson	0.6276 ± 0.0627	0.6276 ± 0.0627	0.6276 ± 0.0627	0.6276 ± 0.0627	0.6276 ± 0.0627	0.6276 ± 0.0627
	Poisson	0.6470 ± 0.0588	0.6470 ± 0.0588	0.6470 ± 0.0588	0.6470 ± 0.0588	0.6470 ± 0.0588	0.6470 ± 0.0588

8.2 Assessing generated data quality

In this experiment, we use synthetic data metrics developed by [Dat, 2025] to quantify the quality of the generated data as implemented by the `sdmetrics` package. We use TCGA COADREAD dataset augmented to class size 5000 to assess the quality. We separate the synthetic and real data from our augmented training dataset and compute the following metrics:

1. Shape: This metric describes the overall column-wise distribution similarity of the two datasets. It uses the complement of the Kolmogorv-Smirnov statistic (KS-statistic) so that a higher score indicates higher similarity between the real and synthetic data.

2. Trend: This metric measures the correlation between a pair of columns and then computes the similarity of this correlation between real and synthetic datasets. A higher score means the pairwise correlations of real and synthetic data are the same.

3. Detection: This metric uses a machine learning model, Logistic Regression in our case, to distinguish between real and synthetic samples. A higher score indicates higher difficulty in distinguishing synthetic from real data.

4. Coverage: This metric measures whether a synthetic column covers the full range of values present in the corresponding real column. A higher score means higher coverage.

All of these metrics are averaged across the 5x5 cross-validation splits and reported in Table 11. All proposed augmentation methods capture the real data distribution, i.e., Shape and Trend, fairly well. Since the inter-class and intra-class methods mix gene blocks between samples, the shapes of the distributions are more similar and so the detection score is also much higher. The inter-class method mixes gene blocks between classes and thus ignores any dependencies between genes that may exist. This is reflected by the relatively lower Trend score compared to intra-class sampling method. The Mod. GP sampling method shows perfect coverage, although the detection score is lower. This is likely due to sampling data from tails of the distribution. The Mod. Poisson method has the lowest coverage and worst detection score. This is likely due to the method not accounting for variance in the data and tending to generate samples around cluster centres, deviating from the real data distribution.

In addition to these metrics, we also compute the silhouette scores using the cosine metric for the real and synthetic data. We compute the silhouette error rate of synthetic data with real data as reference using the formula described in Equation 6, where $\mathbb{E}_{ss}$ is the average synthetic silhouette score and $\mathbb{E}_{sr}$ is the average real silhouette score. The scores are averaged across the 5x5 cross-validation splits and the results are reported in Table 12. We find that our Mod. GP, intra-class and inter-class crossover methods have a lower error rate compared to SMOTE. These results suggest that the sample distance properties are better preserved by these methods compared to state-of-the-art. The Mod. Poisson tends to generate data close to the real samples, not taking into account any variance in the data and hence has a much higher silhouette score.

$$error\_rate = \frac{|\mathbb{E}_{ss} - \mathbb{E}_{sr}|}{\mathbb{E}_{sr}} \tag{6}$$

Table 11: Quality of synthetic data with respect to real data as measured by KS-Statistic for marginal distribution (Shape), correlation similarity between pairs of columns (Trend), difficulty of distinguishing synthetic from real by a machine learning model (Detection), coverage of full range of values present in real columns (Coverage), silhouette score of synthetic and real data. All values shown here are the average across 5x5 cross-validation splits. Higher the better for all metrics.

Sampling Method	Shape	Trend	Detection	Coverage
Mod. Gamma-Poisson	0.852334	0.926452	0.585757	1.000000
Inter-Class	0.876892	0.916918	0.780373	0.999909
Intra-Class	0.864476	0.948716	0.767157	0.999747
Mod. Poisson	0.847289	0.926173	0.243750	0.807247

Table 12: Silhouette scores of synthetic and real data, and the % error rate of synthetic with respect to real. All values shown here are the average across 5x5 cross-validation splits.

Sampling Method	Synthetic Silhouette Score	Real Silhouette Score	Silhouette Error Rate (%)
Mod. Gamma-Poisson	0.182807	0.210522	13.16%
Inter-Class	0.160019	0.210522	23.99%
Intra-Class	0.196591	0.210522	6.62%
Mod. Poisson	0.597982	0.210522	184.04%
SMOTE	0.281503	0.210522	33.72%

8.3 Classification performance

8.3.1 Non-overlapping signatures

Table 13 describes the results of CMS classification of the augmentation methods on in-domain TCGA test data and Table 14 for out-of-domain CPTAC data. Figure 7 and Figure 8 illustrate the distribution of the ROC-AUC scores for the same task across all cross-validation splits for in-domain and out-of-domain test data, respectively. From Table 13, we observe that the Mod. GP augmentation method is best suited for in-domain data. When data is augmented to class size "Max", the average balanced accuracy is 87.57% $\pm$ 0.029, at class size 500 it increases to 88.18% $\pm$ 0.028, and it begins to saturate at class size 5000 with 88.43% $\pm$ 0.029. As expected, the choice of class size impacts the effectiveness of the data augmentation methods. Interestingly, the distribution-based Mod. GP performs best when the number of augmented samples is pushed to the extreme, benefiting from the increased class size in contrast to other methods. However, the trend indicates that over-augmentation offers little to no benefits at the expense of increased computation ($\approx$ 0.3% performance gain from class size 500 to 5000).

To get deeper insights, we additionally compute the confusion matrices for all sampling methods, classifiers, class sizes and cross-validation splits (results for all are provided in a supplemental file) on CPTAC data. Figure 6 shows the confusion matrix for each sampling method averaged across all classifiers and cross-validation splits for class size 500 to provide a general overview of the strengths and weaknesses of each augmentation method. We see that the inter- and intra-class sampling methods improve the classification of CMS1 (+17.8% and +14.5%, respectively) and CMS2 (+17.3% and +4.1%, respectively) classes over unaugmented data. However, the performance in classifying CMS3 (-7.7% and -4%, respectively) and CMS4 (-5.7% and -3.9%, respectively) falls compared to unaugmented data. Similarly, Mod. GP method improves CMS1 performance by +10.4% and CMS3 by +3.2% at the cost of CMS2 (-3.6%) and CMS4 (-1.4%). Mod. Poisson, SMOTE and replacement sampling methods show comparable performance to unaugmented data making the augmentations uninformative.

EPICC evaluation. The EPICC [Heide et al., 2022, Househam et al., 2022] RNA-Seq raw counts, downloaded from `https://data.mendeley.com/datasets/dvv6kf856g/3`, is processed in the same way as CPTAC. The counts are first subset to the 40 colotype genes [Buechler et al., 2020], log2-FPKM normalised and then standardised using the training split's standardisation parameters. Consistent with our findings, we see from Figure 9 that over-augmenting the data provides little benefit and the best performances are achieved at class size 500. All augmentation methods perform significantly better than unaugmented data. Figure 10 shows the confusion matrices and mean balanced accuracy obtained on this dataset for each augmentation method, averaged across all classifiers and cross-validation splits (results are provided in a supplemental file). At class size 500, we see that the intra-class sampling method shows the best generalisation at 67.5%, followed by Mod. GP at 64.5% and inter-class sampling at 62% compared to unaugmented data with 56.2% balanced accuracy. The intra-class method boosts the classification performance of CMS4 by 32.3%, CMS3 by 5.9%, CMS2 by 4.8% and CMS1 by 2.2% over unaugmented data. Similarly, the inter-class method improves classification of CMS4 by 8%, CMS3 by 11.1%, and CMS2 by 4.2%. Although mod. GP shows a higher overall performance compared to inter-class sampling, the inter-class method outperforms it on CMS1, CMS2 and CMS3 classifications. The performance boost of mod. GP comes from the correct classification of 65.9% of CMS4 samples as opposed to only 47% by inter-class sampling.

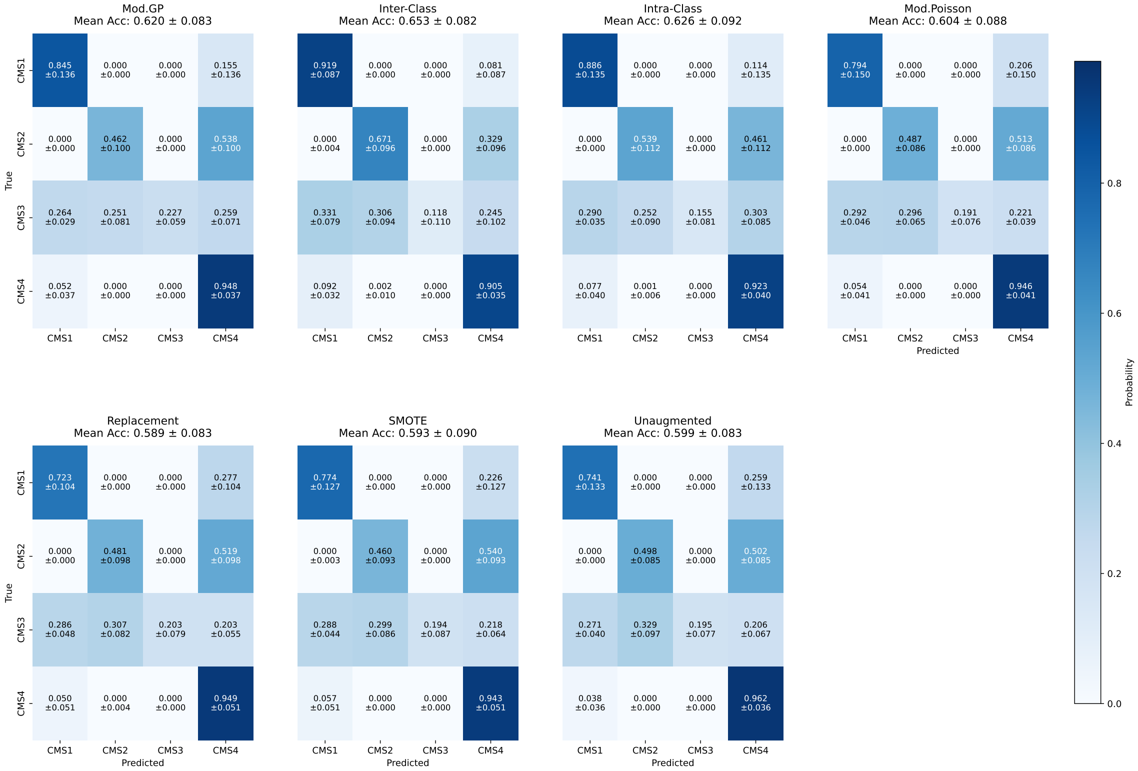

Figure 6: Confusion matrices for external CPTAC data averaged over all classifiers and 5x5 cross-validation splits for each augmentation method, augmented to class size 500. Overall, inter-class sampling method improves performance significantly with approximately 18% gain in predicting CMS1 and +17% gain in predicting CMS2 over unaugmented data.

Table 13: Average balanced accuracy and standard deviation on in-domain TCGA test set from 5x5 cross-validation per classifier model and an average across these models for different augmentation methods applied to the entire training dataset (100%).

Class Size	Sampling Methods	EBM	KNN	Logistic	RF	SVM-RBF	Average
Max	Mod. Gamma-Poisson	0.8841 ± 0.0297	0.8331 ± 0.0325	0.8936 ± 0.0266	0.8782 ± 0.0316	0.8896 ± 0.0253	0.8757 ± 0.0291
	Inter-Class	0.8948 ± 0.0276	0.8465 ± 0.0371	0.8917 ± 0.0292	0.8821 ± 0.0344	0.9011 ± 0.0296	0.8832 ± 0.0316
	Intra-Class	0.8796 ± 0.0244	0.8434 ± 0.0353	0.8837 ± 0.0228	0.8763 ± 0.0317	0.8808 ± 0.0327	0.8728 ± 0.0294
	Mod. Poisson	0.8819 ± 0.0249	0.878 ± 0.0335	0.8885 ± 0.0313	0.8729 ± 0.0377	0.8829 ± 0.0308	0.8808 ± 0.0316
	Replacement	0.8744 ± 0.0328	0.8404 ± 0.04	0.891 ± 0.022	0.8744 ± 0.0288	0.8974 ± 0.0253	0.8755 ± 0.0298
	SMOTE	0.8904 ± 0.0225	0.8666 ± 0.0342	0.8852 ± 0.0306	0.8725 ± 0.0306	0.8965 ± 0.0243	0.8822 ± 0.0284
	Unaugmented	0.8674 ± 0.0264	0.841 ± 0.0354	0.8889 ± 0.0319	0.8615 ± 0.0335	0.8861 ± 0.0315	0.869 ± 0.0317
500	Mod. Gamma-Poisson	0.9003 ± 0.0285	0.8364 ± 0.0368	0.8991 ± 0.0217	0.8777 ± 0.0284	0.8955 ± 0.0264	0.8818 ± 0.0283
	Inter-Class	0.8762 ± 0.0334	0.8319 ± 0.041	0.8765 ± 0.0266	0.8727 ± 0.0275	0.8846 ± 0.0314	0.8684 ± 0.032
	Intra-Class	0.8827 ± 0.0299	0.8485 ± 0.0408	0.8763 ± 0.0245	0.8704 ± 0.0379	0.8873 ± 0.0298	0.873 ± 0.0326
	Mod. Poisson	0.8825 ± 0.027	0.8739 ± 0.0402	0.8858 ± 0.027	0.8651 ± 0.0322	0.885 ± 0.0285	0.8784 ± 0.031
	Replacement	0.8684 ± 0.0347	0.8226 ± 0.0477	0.8792 ± 0.0297	0.8728 ± 0.0322	0.8887 ± 0.0309	0.8663 ± 0.035
	SMOTE	0.8877 ± 0.0264	0.8602 ± 0.0406	0.888 ± 0.0315	0.8711 ± 0.0324	0.8938 ± 0.0261	0.8801 ± 0.0314
	Unaugmented	0.8674 ± 0.0264	0.841 ± 0.0354	0.8889 ± 0.0319	0.8615 ± 0.0335	0.8861 ± 0.0315	0.869 ± 0.0317
5000	Mod. Gamma-Poisson	0.8927 ± 0.0291	0.8583 ± 0.0356	0.8955 ± 0.0256	0.8781 ± 0.0353	0.897 ± 0.0238	0.8843 ± 0.0299
	Inter-Class	0.8445 ± 0.0351	0.837 ± 0.0403	0.8664 ± 0.0298	0.8479 ± 0.0378	0.8649 ± 0.0297	0.8521 ± 0.0345
	Intra-Class	0.8603 ± 0.0281	0.8472 ± 0.0465	0.8668 ± 0.0278	0.8636 ± 0.0377	0.8723 ± 0.0257	0.8621 ± 0.0332
	Mod. Poisson	0.8832 ± 0.0282	0.8688 ± 0.0383	0.8939 ± 0.0276	0.8674 ± 0.0352	0.8893 ± 0.0272	0.8805 ± 0.0313
	Replacement	0.8654 ± 0.0346	0.8048 ± 0.045	0.8669 ± 0.0349	0.8622 ± 0.0374	0.8849 ± 0.0267	0.8568 ± 0.0357
	SMOTE	0.8812 ± 0.033	0.8464 ± 0.0405	0.8776 ± 0.0296	0.8668 ± 0.0346	0.8927 ± 0.0292	0.8729 ± 0.0334
	Unaugmented	0.8674 ± 0.0264	0.841 ± 0.0354	0.8889 ± 0.0319	0.8615 ± 0.0335	0.8861 ± 0.0315	0.869 ± 0.0317

Table 14: Average balanced accuracy and standard deviation on out-of-domain CPTAC test set from 5x5 cross-validation with different classifier models and an overall average of these models for different augmentation methods. The entire training dataset (100%) was used for augmentation.

Class Size	Sampling Methods	EBM	KNN	Logistic	RF	SVM-RBF	Average
Max	Mod. Gamma-Poisson	0.6506 ± 0.0334	0.5781 ± 0.0297	0.5396 ± 0.0513	0.6348 ± 0.0248	0.6025 ± 0.029	0.6011 ± 0.0336
	Inter-Class	0.67 ± 0.0342	0.5876 ± 0.036	0.5179 ± 0.0523	0.6306 ± 0.0181	0.599 ± 0.0268	0.601 ± 0.0335
	Intra-Class	0.6474 ± 0.0299	0.5947 ± 0.0374	0.509 ± 0.0501	0.6436 ± 0.0193	0.5986 ± 0.0253	0.5986 ± 0.0324
	Mod. Poisson	0.6332 ± 0.0318	0.6225 ± 0.026	0.537 ± 0.0432	0.6309 ± 0.0401	0.6013 ± 0.0372	0.605 ± 0.0357
	Replacement	0.6314 ± 0.0331	0.5822 ± 0.0311	0.55 ± 0.0302	0.6369 ± 0.032	0.5838 ± 0.0367	0.5969 ± 0.0326
	SMOTE	0.6375 ± 0.0353	0.5961 ± 0.0333	0.5361 ± 0.0323	0.6557 ± 0.0274	0.5788 ± 0.0347	0.6009 ± 0.0326
	Unaugmented	0.637 ± 0.0252	0.5917 ± 0.0257	0.5347 ± 0.0337	0.6256 ± 0.0354	0.6058 ± 0.0285	0.599 ± 0.0297
500	Mod. Gamma-Poisson	0.6732 ± 0.0334	0.6 ± 0.0346	0.5681 ± 0.0485	0.654 ± 0.0196	0.6055 ± 0.0313	0.6202 ± 0.0335
	Inter-Class	0.7135 ± 0.0242	0.6359 ± 0.034	0.6213 ± 0.0346	0.6657 ± 0.0329	0.6303 ± 0.0345	0.6534 ± 0.032
	Intra-Class	0.6705 ± 0.0281	0.6145 ± 0.0355	0.5646 ± 0.0475	0.6771 ± 0.0206	0.6017 ± 0.0253	0.6257 ± 0.0314
	Mod. Poisson	0.6256 ± 0.0314	0.6402 ± 0.03	0.5313 ± 0.026	0.6353 ± 0.033	0.5892 ± 0.0418	0.6043 ± 0.0324
	Replacement	0.6255 ± 0.0261	0.5732 ± 0.036	0.5531 ± 0.0361	0.6275 ± 0.024	0.5673 ± 0.0343	0.5893 ± 0.0313
	SMOTE	0.616 ± 0.0343	0.6009 ± 0.0378	0.5339 ± 0.0268	0.6491 ± 0.0309	0.5642 ± 0.0344	0.5928 ± 0.0328
	Unaugmented	0.637 ± 0.0252	0.5917 ± 0.0257	0.5347 ± 0.0337	0.6256 ± 0.0354	0.6058 ± 0.0285	0.599 ± 0.0297
5000	Mod. Gamma-Poisson	0.6845 ± 0.0145	0.6253 ± 0.0287	0.6344 ± 0.0197	0.6775 ± 0.0131	0.6243 ± 0.0284	0.6492 ± 0.0209
	Inter-Class	0.6532 ± 0.0183	0.6748 ± 0.0253	0.6533 ± 0.0153	0.6821 ± 0.0208	0.6592 ± 0.0154	0.6645 ± 0.019
	Intra-Class	0.6637 ± 0.0421	0.6367 ± 0.0299	0.5917 ± 0.038	0.673 ± 0.023	0.605 ± 0.0279	0.634 ± 0.0322
	Mod. Poisson	0.6243 ± 0.0222	0.6354 ± 0.0192	0.5293 ± 0.023	0.6057 ± 0.0252	0.571 ± 0.0358	0.5932 ± 0.0251
	Replacement	0.6271 ± 0.0353	0.5725 ± 0.0276	0.5588 ± 0.0518	0.6206 ± 0.0269	0.58 ± 0.0374	0.5918 ± 0.0358
	SMOTE	0.6228 ± 0.0325	0.5842 ± 0.0274	0.5336 ± 0.0412	0.6139 ± 0.0324	0.5709 ± 0.041	0.5851 ± 0.0349
	Unaugmented	0.637 ± 0.0252	0.5917 ± 0.0257	0.5347 ± 0.0337	0.6256 ± 0.0354	0.6058 ± 0.0285	0.599 ± 0.0297

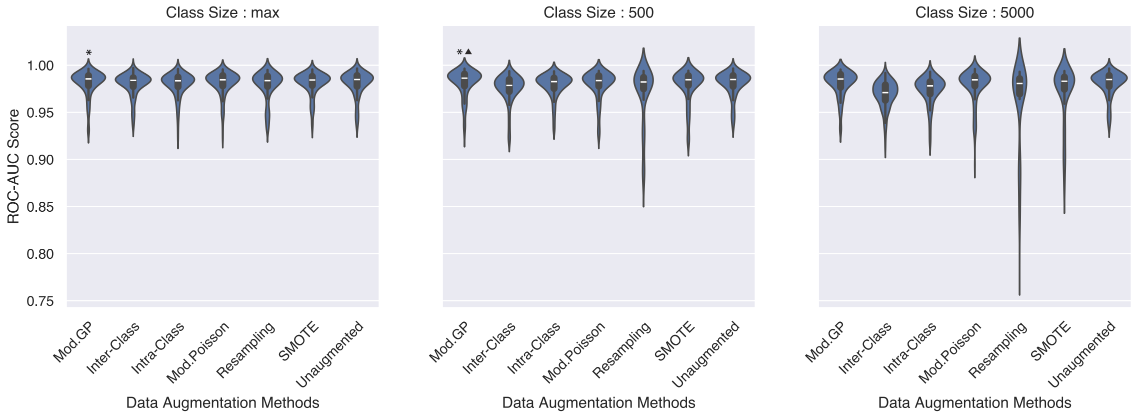

Figure 7: Distribution of ROC-AUC scores on in-domain TCGA test set across all classification models (EBM, KNN, Logistic, RF, SVM-RBF) for each augmentation method and class size. The trend indicates that as augmentation size increases, Mod. GP tends to achieve a higher score. $*$ indicates that the method is significantly better than unaugmented data. ▲ indicates that the method is significantly better than all other methods.

SMOTE shows a 3% overall performance increase over unaugmented data. While it improves classification performance of CMS1 (+4.1%), CMS2 (+4.7%) and CMS3 (+5%), it fails to generalise to CMS4 (-1.2%). Detailed performance per classifier model is shown in Table 15. We also show the ROC-AUC scores averaged across classifiers for the three class sizes as seen in Figure 11 where the intra-class and Mod. GP methods are significantly better than unaugmented data.

8.3.2 Overlapping signatures

Figure 12 illustrates the distribution of balanced accuracy scores for PAM50 subtype classification across GSE20713 cross-validation test splits. Figure 13 and Figure 14 illustrate the distribution of the ROC-AUC scores across cross-validation splits for in-domain and out-of-domain test data, respectively. Table 16 and Table 17 describe the average balanced accuracy scores on in-domain GSE20713 and out-of-domain METABRIC test sets for each classifier and augmentation method. In addition to Mod. GP, we see that at lower augmentation sizes both the modified and unmodified Poisson sampling methods are associated with best performance. The negligible performance differences between these two methods could be attributed to the low number of both real samples and the number of generated samples, which limits the diversity of the generated samples. Although the Mod. Poisson sampling method sometimes performs on par, particularly in the lower augmentation size regime, the Mod. GP should be the preferred method as it can handle over-dispersion.

To get further insights on which classes benefit from the augmentation, we compute confusion matrices for all classifiers and augmentation methods for the 25 cross-validation splits (results for all are provided in a supplemental file). Since class size 50 is the best performing, we illustrate the average confusion matrix for each augmentation method for this class averaged across all classifiers and cross-validation splits in Figure 15. Since the classifiers considered are a mix of linear and non-linear models, we get a global picture of which classes benefit from which augmentation methods.

Table 15: Average balanced accuracy and standard deviation on out-of-domain EPICC test set from 5x5 cross-validation with different classifier models and an overall average of these models for different augmentation methods. The entire training dataset (100%) was used for augmentation.

Class Size	Sampling Methods	EBM	KNN	Logistic	RF	SVM-RBF	Average
max	Mod. Gamma-Poisson	0.7028 ± 0.0594	0.4249 ± 0.0291	0.7033 ± 0.0583	0.6833 ± 0.0406	0.4162 ± 0.0398	0.5861 ± 0.0454
	Inter-Class	0.7090 ± 0.0625	0.4477 ± 0.0345	0.7307 ± 0.0575	0.7056 ± 0.0665	0.4107 ± 0.0666	0.6007 ± 0.0575
	Intra-Class	0.7808 ± 0.0619	0.4376 ± 0.0294	0.7776 ± 0.0548	0.7239 ± 0.0547	0.4311 ± 0.0893	0.6302 ± 0.0580
	Mod. Poisson	0.7547 ± 0.0527	0.4621 ± 0.0238	0.6738 ± 0.0443	0.6994 ± 0.0585	0.2857 ± 0.0749	0.5751 ± 0.0508
	Replacement	0.7676 ± 0.0556	0.4337 ± 0.0359	0.6864 ± 0.0503	0.6981 ± 0.0672	0.3557 ± 0.0728	0.5883 ± 0.0564
	SMOTE	0.7769 ± 0.0492	0.4490 ± 0.0356	0.6829 ± 0.0521	0.6943 ± 0.0670	0.3004 ± 0.0563	0.5807 ± 0.0520
	Unaugmented	0.7553 ± 0.0390	0.4737 ± 0.0184	0.6481 ± 0.0477	0.6277 ± 0.0618	0.3044 ± 0.0461	0.5618 ± 0.0426
500	Mod. Gamma-Poisson	0.7774 ± 0.0420	0.4637 ± 0.0384	0.7422 ± 0.0540	0.7848 ± 0.0364	0.4581 ± 0.1141	0.6453 ± 0.0570
	Inter-Class	0.6860 ± 0.0641	0.5002 ± 0.0411	0.7143 ± 0.0380	0.7118 ± 0.0581	0.4893 ± 0.0839	0.6203 ± 0.0570
	Intra-Class	0.8318 ± 0.0410	0.5147 ± 0.0527	0.7626 ± 0.0508	0.8108 ± 0.0580	0.4558 ± 0.0974	0.6751 ± 0.0600
	Mod. Poisson	0.7567 ± 0.0429	0.4832 ± 0.0216	0.6785 ± 0.0553	0.7129 ± 0.0626	0.2719 ± 0.0433	0.5806 ± 0.0451
	Replacement	0.7763 ± 0.0506	0.4859 ± 0.0312	0.6776 ± 0.0495	0.7401 ± 0.0561	0.3427 ± 0.0721	0.6045 ± 0.0519
	SMOTE	0.7674 ± 0.0416	0.4700 ± 0.0250	0.6907 ± 0.0543	0.7148 ± 0.0549	0.3241 ± 0.0641	0.5934 ± 0.0480
	Unaugmented	0.7553 ± 0.0390	0.4737 ± 0.0184	0.6481 ± 0.0477	0.6277 ± 0.0618	0.3044 ± 0.0461	0.5618 ± 0.0426
5000	Mod. Gamma-Poisson	0.8222 ± 0.0314	0.5361 ± 0.0409	0.7771 ± 0.0262	0.8129 ± 0.0294	0.4390 ± 0.1094	0.6775 ± 0.0475
	Inter-Class	0.6734 ± 0.0355	0.5721 ± 0.0440	0.6810 ± 0.0312	0.7293 ± 0.0414	0.5055 ± 0.0657	0.6323 ± 0.0436
	Intra-Class	0.7799 ± 0.0490	0.5686 ± 0.0456	0.7319 ± 0.0339	0.8517 ± 0.0312	0.4594 ± 0.0761	0.6783 ± 0.0472
	Mod. Poisson	0.7490 ± 0.0455	0.5016 ± 0.0210	0.6902 ± 0.0507	0.7164 ± 0.0483	0.2733 ± 0.0275	0.5861 ± 0.0386
	Replacement	0.7764 ± 0.0451	0.4922 ± 0.0241	0.6468 ± 0.0593	0.7406 ± 0.0537	0.3180 ± 0.0496	0.5948 ± 0.0464
	SMOTE	0.7539 ± 0.0414	0.4901 ± 0.0195	0.6761 ± 0.0628	0.7471 ± 0.0520	0.3071 ± 0.0570	0.5949 ± 0.0465
	Unaugmented	0.7553 ± 0.0390	0.4737 ± 0.0184	0.6481 ± 0.0477	0.6277 ± 0.0618	0.3044 ± 0.0461	0.5618 ± 0.0426

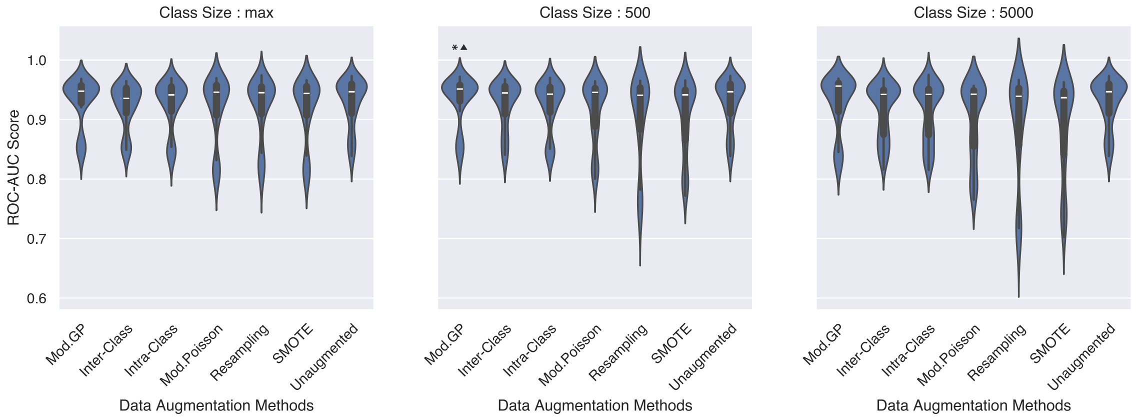

Figure 8: Distribution of ROC-AUC scores on out-of-domain CPTAC test set across all classification models (EBM, KNN, Logistic, RF, SVM-RBF) for each augmentation method and class size. Replacement, SMOTE and Mod. Poisson are significantly worse than the other methods, including unaugmented, in all class sizes. $*$ indicates that the method is significantly better than unaugmented data. ▲ indicates that the method is significantly better than all other methods.

From Figure 15, we see that Mod. GP and and Mod. Poisson are the only methods handling overlapping genes the best, with a significant performance improvement over unaugmented data. The performance gains come primarily from classifying Luminal B samples correctly, with over 30% increase over unaugmented data, and Luminal A with 6-8% increase over unaugmented data. The poor performance of the inter-class sampling method further supports the inference that this method cannot naïvely handle overlapping genes as classifiers trained on this data tend to classify most of the samples as "Basal" type. Intra-class sampling is comparatively better suited for overlapping genes than inter-class sampling and shows similar performance to SMOTE.

8.4 Effect of 10% real data

Results on classification performance when only 10% of the real colorectal cancer data are provided is shown in Table 18 for in-domain testing (TCGA) and Table 19 for out-of-domain testing (CPTAC).

8.5 VAE generated sample classification

Figure 16 illustrates the distribution of balanced accuracy of CMS classification on VAE generated samples modelled on TCGA test data and Table 20 quantifies the average balanced accuracy for each classification model and augmentation method. Similarly, Table 21 quantifies the average balanced accuracy for each classification model and augmentation method for VAE generated samples modeled on CPTAC test data. As expected from in-domain testing, augmentation is absolutely necessary in this low-data regime to learn anything useful from the data. On average (see Figure 16), almost all methods are on par with each other, with an average difference of around 2% between the methods. The Mod. GP augmentation method achieves the best performances with

Table 16: Average balanced accuracy scores and standard deviation on in-domain GSE20713 test set from 5x5 cross-validation for supervised classification of PAM50 subtype prediction with different classifier models and an overall average over these models for the different augmentation methods. Results indicate that augmentation to class size 50 results in Mod. GP and Mod. Poisson performing significantly better than unaugmented data at a significance threshold of 0.05. Further increasing the class size to 500 results in only the Mod. Poisson performing significantly better than unaugmented data. Note that the test set size here is $\approx \mathbf{14-15}$ samples.

Class Size	Sampling Method	EBM	KNN	Logistic	RF	SVM-RBF	Average
Max	Mod. Gamma-Poisson	0.71 ± 0.083	0.69 ± 0.101	0.66 ± 0.152	0.75 ± 0.116	0.77 ± 0.095	0.72 ± 0.109
	Inter-Class	0.711 ± 0.099	0.703 ± 0.096	0.692 ± 0.132	0.773 ± 0.089	0.749 ± 0.097	0.726 ± 0.102
	Intra-Class	0.743 ± 0.118	0.713 ± 0.089	0.671 ± 0.139	0.771 ± 0.097	0.737 ± 0.100	0.727 ± 0.109
	Mod. Poisson	0.729 ± 0.091	0.695 ± 0.107	0.671 ± 0.136	0.755 ± 0.128	0.767 ± 0.086	0.723 ± 0.110
	Resampling	0.735 ± 0.101	0.686 ± 0.115	0.669 ± 0.130	0.769 ± 0.117	0.746 ± 0.099	0.721 ± 0.113
	SMOTE	0.739 ± 0.110	0.697 ± 0.100	0.669 ± 0.131	0.743 ± 0.114	0.754 ± 0.096	0.721 ± 0.110
	Gamma-Poisson	0.734 ± 0.100	0.702 ± 0.119	0.686 ± 0.134	0.760 ± 0.090	0.764 ± 0.100	0.729 ± 0.109
	Poisson	0.731 ± 0.107	0.710 ± 0.103	0.669 ± 0.128	0.757 ± 0.116	0.755 ± 0.090	0.724 ± 0.109
	Unaugmented	0.727 ± 0.116	0.703 ± 0.100	0.663 ± 0.138	0.759 ± 0.113	0.741 ± 0.107	0.719 ± 0.115
50	Mod. Gamma-Poisson	0.731 ± 0.108	0.740 ± 0.111	0.757 ± 0.112	0.763 ± 0.088	0.739 ± 0.106	0.746 ± 0.105
	Inter-Class	0.656 ± 0.106	0.693 ± 0.098	0.621 ± 0.113	0.665 ± 0.144	0.696 ± 0.105	0.666 ± 0.113
	Intra-Class	0.712 ± 0.119	0.729 ± 0.114	0.685 ± 0.133	0.757 ± 0.115	0.739 ± 0.120	0.724 ± 0.120
	Mod. Poisson	0.728 ± 0.096	0.740 ± 0.132	0.751 ± 0.129	0.751 ± 0.090	0.743 ± 0.103	0.742 ± 0.110
	Resampling	0.691 ± 0.114	0.685 ± 0.104	0.653 ± 0.120	0.745 ± 0.122	0.709 ± 0.122	0.697 ± 0.116
	SMOTE	0.725 ± 0.072	0.689 ± 0.100	0.650 ± 0.142	0.755 ± 0.111	0.706 ± 0.127	0.705 ± 0.110
	Gamma-Poisson	0.712 ± 0.102	0.705 ± 0.099	0.711 ± 0.116	0.715 ± 0.107	0.758 ± 0.113	0.720 ± 0.107
	Poisson	0.743 ± 0.117	0.728 ± 0.140	0.753 ± 0.106	0.742 ± 0.116	0.772 ± 0.115	0.748 ± 0.119
	Unaugmented	0.727 ± 0.116	0.703 ± 0.100	0.663 ± 0.138	0.759 ± 0.113	0.741 ± 0.107	0.719 ± 0.115
500	Mod. Gamma-Poisson	0.702 ± 0.109	0.737 ± 0.112	0.765 ± 0.114	0.751 ± 0.094	0.737 ± 0.121	0.739 ± 0.110
	Inter-Class	0.598 ± 0.094	0.687 ± 0.097	0.622 ± 0.106	0.628 ± 0.104	0.682 ± 0.086	0.643 ± 0.098
	Intra-Class	0.676 ± 0.108	0.745 ± 0.097	0.721 ± 0.134	0.747 ± 0.118	0.740 ± 0.115	0.726 ± 0.115
	Mod. Poisson	0.766 ± 0.091	0.750 ± 0.141	0.772 ± 0.112	0.757 ± 0.101	0.751 ± 0.119	0.759 ± 0.113
	Resampling	0.690 ± 0.105	0.691 ± 0.112	0.639 ± 0.128	0.722 ± 0.111	0.651 ± 0.117	0.679 ± 0.115
	SMOTE	0.645 ± 0.132	0.675 ± 0.103	0.634 ± 0.129	0.716 ± 0.120	0.631 ± 0.127	0.660 ± 0.122
	Gamma-Poisson	0.715 ± 0.107	0.755 ± 0.110	0.682 ± 0.136	0.711 ± 0.111	0.720 ± 0.131	0.717 ± 0.119
	Poisson	0.722 ± 0.118	0.737 ± 0.120	0.783 ± 0.116	0.769 ± 0.111	0.741 ± 0.114	0.750 ± 0.116
	Unaugmented	0.727 ± 0.116	0.703 ± 0.100	0.663 ± 0.138	0.759 ± 0.113	0.741 ± 0.107	0.719 ± 0.115

Table 17: Average balanced accuracy scores and standard deviation on out-of-domain METABRIC test set from 5x5 cross-validation for supervised classification of PAM50 subtype prediction with different classifier models and an overall average over these models for the different augmentation methods. Results indicate that increasing the augmentation size results in only the Mod. GP and Mod. Poisson methods performing significantly better than unaugmented data at a significance threshold of 0.05.

Class Size	Sampling Method	EBM	KNN	Logistic	RF	SVM-RBF	Average
max	Mod. Gamma-Poisson	0.7175 ± 0.0496	0.6677 ± 0.0717	0.5809 ± 0.1060	0.7146 ± 0.0415	0.3370 ± 0.0157	0.604 ± 0.0569
	Inter-Class	0.7133 ± 0.0443	0.5819 ± 0.0723	0.5560 ± 0.1138	0.7204 ± 0.0391	0.3333 ± 0.0000	0.581 ± 0.0539
	Intra-Class	0.7223 ± 0.0286	0.6109 ± 0.0624	0.5756 ± 0.1138	0.7260 ± 0.0309	0.3333 ± 0.0000	0.594 ± 0.0471
	Mod. Poisson	0.7138 ± 0.0518	0.6470 ± 0.0762	0.6192 ± 0.0902	0.7090 ± 0.0333	0.3423 ± 0.0438	0.606 ± 0.0591
	Resampling	0.7237 ± 0.0384	0.5952 ± 0.0670	0.5881 ± 0.0970	0.7270 ± 0.0327	0.3333 ± 0.0000	0.593 ± 0.0470
	SMOTE	0.7388 ± 0.0241	0.5856 ± 0.0739	0.6011 ± 0.0970	0.7121 ± 0.0328	0.3333 ± 0.0000	0.594 ± 0.0456
	Gamma-Poisson	0.717 ± 0.054	0.685 ± 0.069	0.597 ± 0.087	0.724 ± 0.032	0.342 ± 0.029	0.613 ± 0.054
	Poisson	0.721 ± 0.036	0.647 ± 0.084	0.584 ± 0.108	0.717 ± 0.036	0.335 ± 0.006	0.601 ± 0.054
	Unaugmented	0.7412 ± 0.0273	0.6195 ± 0.0521	0.5923 ± 0.1219	0.7361 ± 0.0340	0.3333 ± 0.0000	0.604 ± 0.0471
50	Mod. Gamma-Poisson	0.7053 ± 0.0640	0.7563 ± 0.0343	0.6725 ± 0.0756	0.7155 ± 0.0573	0.7437 ± 0.0532	0.719 ± 0.0569
	Inter-Class	0.5005 ± 0.0734	0.4607 ± 0.0689	0.4509 ± 0.1010	0.5532 ± 0.0755	0.3333 ± 0.0000	0.460 ± 0.0638
	Intra-Class	0.7254 ± 0.0328	0.5329 ± 0.0806	0.6326 ± 0.1050	0.7048 ± 0.0556	0.3333 ± 0.0000	0.586 ± 0.0548
	Mod. Poisson	0.7237 ± 0.0416	0.7375 ± 0.0299	0.6976 ± 0.0602	0.7364 ± 0.0400	0.7544 ± 0.0580	0.730 ± 0.0459
	Resampling	0.7113 ± 0.0242	0.5255 ± 0.0891	0.5709 ± 0.1077	0.7212 ± 0.0336	0.3333 ± 0.0000	0.572 ± 0.0509
	SMOTE	0.7172 ± 0.0263	0.5544 ± 0.0813	0.5714 ± 0.1082	0.7037 ± 0.0412	0.3333 ± 0.0000	0.576 ± 0.0514
	Gamma-Poisson	0.662 ± 0.073	0.754 ± 0.037	0.609 ± 0.105	0.714 ± 0.049	0.723 ± 0.070	0.692 ± 0.067
	Poisson	0.714 ± 0.057	0.747 ± 0.030	0.684 ± 0.080	0.723 ± 0.058	0.750 ± 0.075	0.724 ± 0.060
	Unaugmented	0.7412 ± 0.0273	0.6195 ± 0.0521	0.5923 ± 0.1219	0.7361 ± 0.0340	0.3333 ± 0.0000	0.604 ± 0.0471
500	Mod. Gamma-Poisson	0.7312 ± 0.0440	0.7536 ± 0.0331	0.7697 ± 0.0429	0.7237 ± 0.0519	0.7600 ± 0.0540	0.748 ± 0.0452
	Inter-Class	0.4097 ± 0.0515	0.4100 ± 0.0627	0.4264 ± 0.0845	0.4273 ± 0.0656	0.3333 ± 0.0000	0.401 ± 0.0529
	Intra-Class	0.7207 ± 0.0362	0.4704 ± 0.0703	0.6048 ± 0.0858	0.5403 ± 0.0839	0.3333 ± 0.0000	0.534 ± 0.0552
	Mod. Poisson	0.7308 ± 0.0359	0.7471 ± 0.0345	0.7644 ± 0.0519	0.7405 ± 0.0386	0.7553 ± 0.0521	0.748 ± 0.0426
	Resampling	0.6956 ± 0.0423	0.5217 ± 0.0748	0.5517 ± 0.0927	0.7134 ± 0.0340	0.3333 ± 0.0000	0.563 ± 0.0488
	SMOTE	0.6883 ± 0.0534	0.5183 ± 0.0776	0.5644 ± 0.0880	0.6578 ± 0.0557	0.3333 ± 0.0000	0.552 ± 0.0549
	Gamma-Poisson	0.681 ± 0.067	0.753 ± 0.034	0.635 ± 0.101	0.664 ± 0.075	0.658 ± 0.087	0.678 ± 0.073
	Poisson	0.739 ± 0.051	0.749 ± 0.029	0.775 ± 0.045	0.732 ± 0.057	0.770 ± 0.037	0.753 ± 0.044
	Unaugmented	0.7412 ± 0.0273	0.6195 ± 0.0521	0.5923 ± 0.1219	0.7361 ± 0.0340	0.3333 ± 0.0000	0.604 ± 0.0471

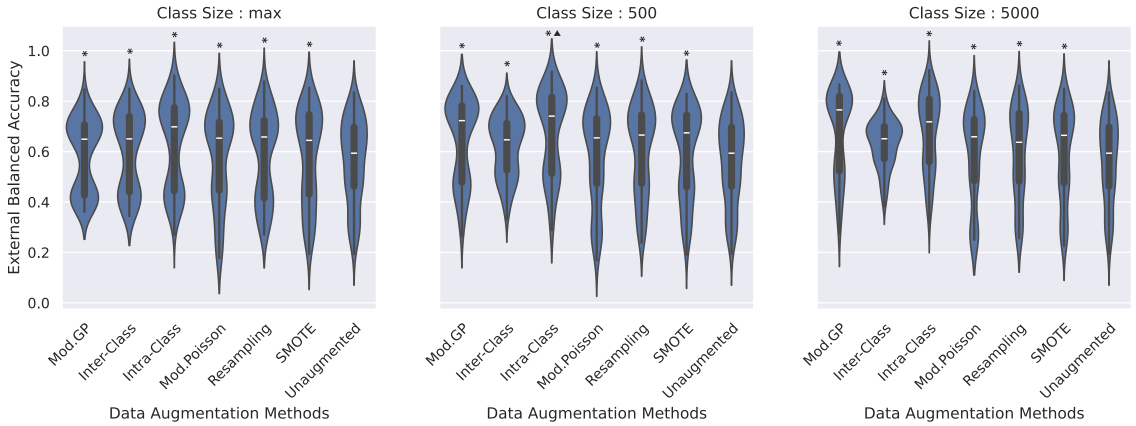

Figure 9: Distribution of balanced accuracy on out-of-domain EPICC test set across all classification models (EBM, KNN, Logistic, RF, SVM-RBF) for each augmentation method, class size and cross-validation split. The trend indicates that intra-class crossover method achieves the best generalisation performance and is a good candidate for tasks needing generalisation as it can achieve the best accuracy with fewer augmented samples compared to Mod. GP. $*$ indicates that the method is significantly better than unaugmented data. ▲ indicates that the method is significantly better than all other methods.

the classification models, with an average accuracy of $77.71\% \pm 0.0536$. All methods are significantly better than the unaugmented case.

8.6 Predicting clinical variables

Table 22 and Table 23 show results for in-domain TCGA test data and out-of-domain CPTAC data, respectively, of different classifier models in predicting the MSI status from VAE embeddings quantified as average balanced accuracy. Table 24 shows the average balanced accuracy on in-domain TCGA test data of different classifier models in predicting the CIMP status from VAE embeddings.

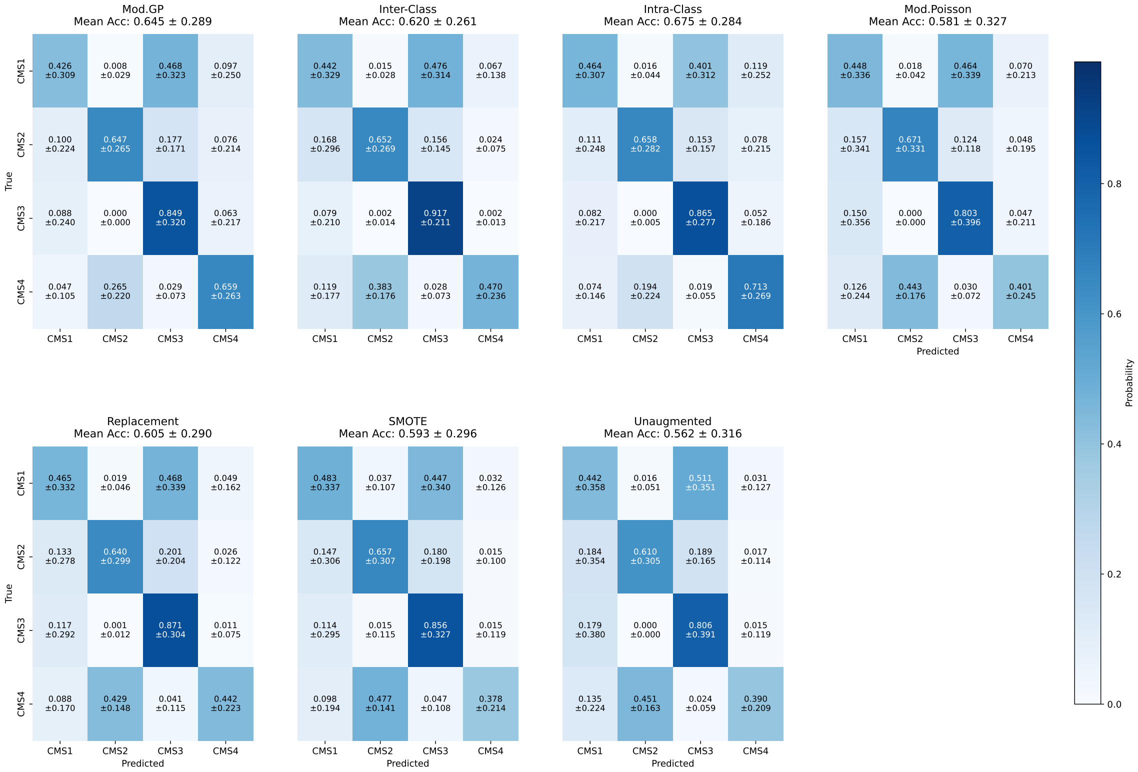

Figure 10: Confusion matrices for external EPICC data averaged over all classifiers and 5x5 cross-validation splits for each augmentation method, augmented to class size 500. Overall, intra-class sampling method improves performance significantly with approximately 32% gain in predicting CMS4, 6% gain in predicting CMS3, 4% gain in predicting CMS2 and 2% gain in predicting CMS1 over unaugmented data.

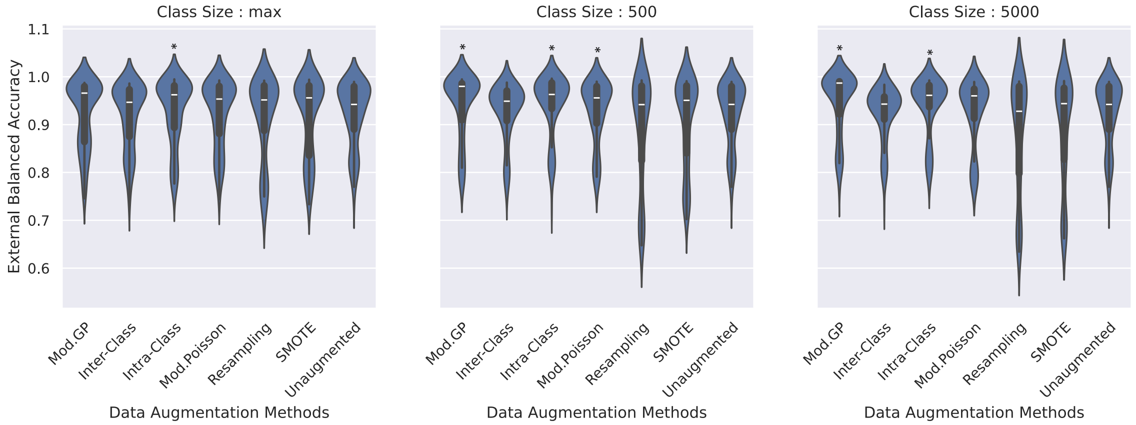

Figure 11: Distribution of ROC-AUC scores on out-of-domain EPICC test set across all classification models (EBM, KNN, Logistic, RF, SVM-RBF) for each augmentation method and class size. $*$ indicates the methods are significantly better than unaugmented.

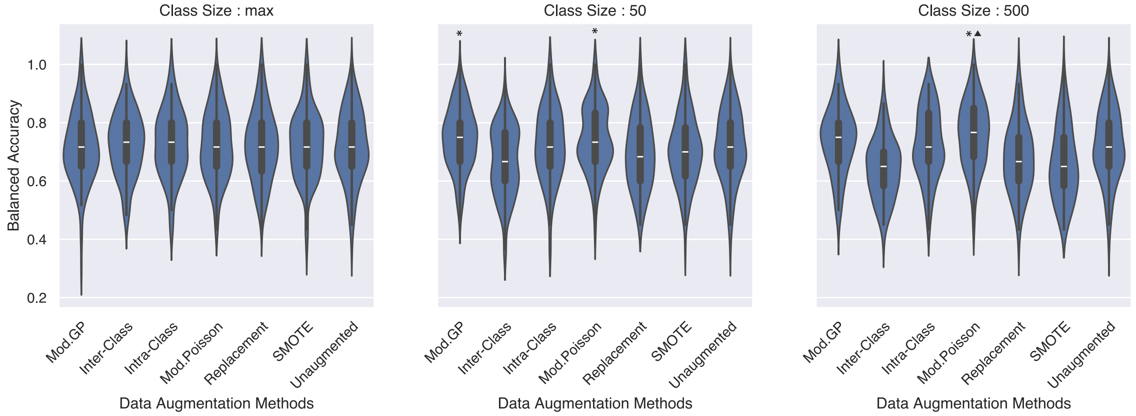

Figure 12: Distribution of balanced accuracy in the case of overlapping gene signatures on in-domain GSE20713 dataset across all classification models (EBM, KNN, Logistic, RF, SVM-RBF) for every augmentation method, class size and cross-validation split. $*$ indicates the methods are significantly better than unaugmented. ▲ indicates that the methods are significantly better than all other methods.

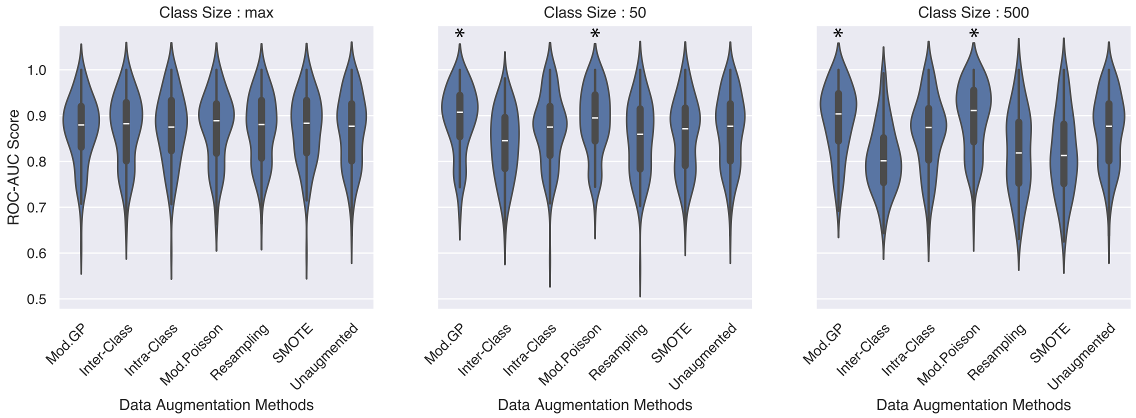

Figure 13: Distribution of ROC-AUC scores on in-domain GSE20713 test set across all classification models (EBM, KNN, Logistic, RF, SVM-RBF) for each augmentation method and class size. The trend indicates that as augmentation size increases, the Mod. GP and Mod. Poisson methods tend to achieve a higher score, while methods like inter-class crossover, SMOTE and replacement sampling perform worse. $*$ indicates that the method is significantly better than the unaugmented case.

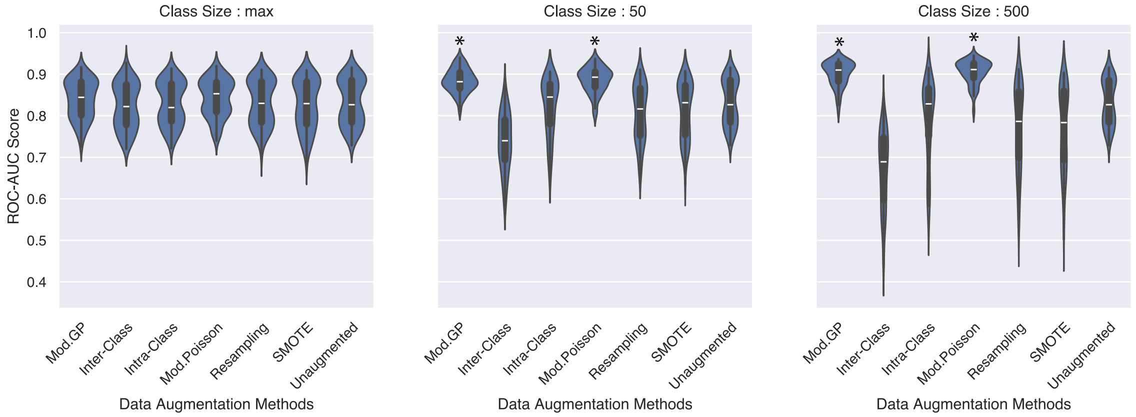

Figure 14: Distribution of ROC-AUC scores on out-of-domain METABRIC test set across all classification models (EBM, KNN, Logistic, RF, SVM-RBF) for each augmentation method and class size. The trend indicates that as augmentation size increases, the Mod. GP and Mod. Poisson methods tend to achieve a higher score, while methods like inter-class crossover, SMOTE and replacement sampling perform worse. $*$ indicates that the method is significantly better than the unaugmented case.

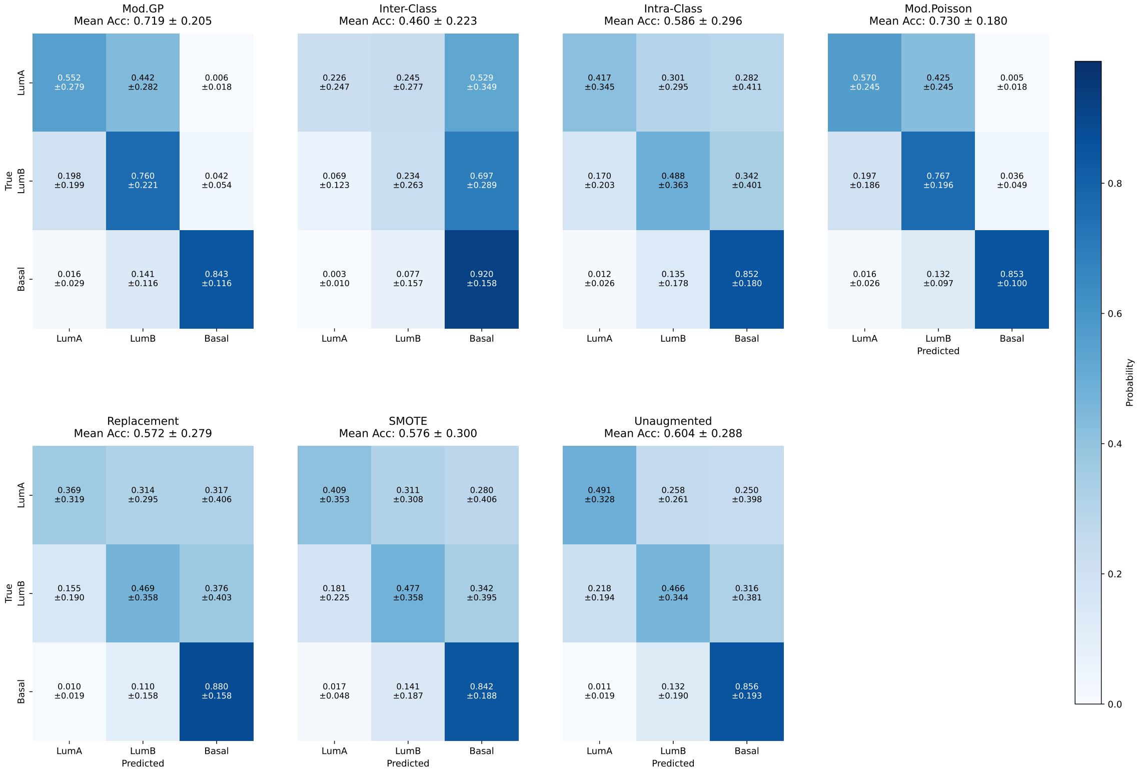

Figure 15: Confusion matrices for external METABRIC data averaged over all classifiers and 5x5 cross-validation splits for each augmentation method, augmented to class size 50. Mod. GP and and Mod. Poisson are the only methods handling overlapping genes the best, with a significant performance improvement over unaugmented data.

Table 18: Average balanced accuracy and standard deviation on in-domain TCGA test set from 5x5 cross-validation with different classifier models and an overall average of these models for different augmentation methods. A portion of the training dataset (10%) was used for augmentation.

Class Size	Sampling Methods	EBM	KNN	Logistic	RF	SVM-RBF	Average
Max	Mod. Gamma-Poisson	0.8126 ± 0.0455	0.7525 ± 0.0542	0.8363 ± 0.0609	0.7914 ± 0.0684	0.8182 ± 0.0567	0.8022 ± 0.0572
	Inter-Class	0.8092 ± 0.0536	0.7666 ± 0.0463	0.8349 ± 0.058	0.8129 ± 0.0433	0.8258 ± 0.0581	0.8099 ± 0.0518
	Intra-Class	0.8141 ± 0.0524	0.7697 ± 0.0535	0.8272 ± 0.0589	0.7973 ± 0.0568	0.822 ± 0.0554	0.8061 ± 0.0554
	Mod. Poisson	0.8049 ± 0.0593	0.8191 ± 0.0418	0.8323 ± 0.0564	0.7838 ± 0.0682	0.7965 ± 0.0593	0.8073 ± 0.057
	Replacement	0.7983 ± 0.052	0.7467 ± 0.0628	0.836 ± 0.0566	0.7842 ± 0.0533	0.8097 ± 0.0563	0.795 ± 0.0562
	SMOTE	0.806 ± 0.0513	0.7827 ± 0.0534	0.835 ± 0.0559	0.7918 ± 0.0659	0.8203 ± 0.0562	0.8072 ± 0.0566
	Unaugmented	0.7707 ± 0.0551	0.7494 ± 0.062	0.8165 ± 0.0561	0.7858 ± 0.047	0.7863 ± 0.0509	0.7817 ± 0.0542
500	Mod. Gamma-Poisson	0.8341 ± 0.0497	0.8012 ± 0.0521	0.8433 ± 0.0498	0.8023 ± 0.0633	0.8412 ± 0.0492	0.8244 ± 0.0528
	Inter-Class	0.789 ± 0.051	0.7889 ± 0.0414	0.7867 ± 0.0522	0.7851 ± 0.0483	0.8116 ± 0.0483	0.7923 ± 0.0482
	Intra-Class	0.7824 ± 0.0479	0.7856 ± 0.0519	0.7974 ± 0.0488	0.7521 ± 0.0571	0.7849 ± 0.0519	0.7805 ± 0.0515
	Mod. Poisson	0.822 ± 0.0589	0.8076 ± 0.0542	0.8463 ± 0.0449	0.7724 ± 0.0533	0.6977 ± 0.0999	0.7892 ± 0.0622
	Replacement	0.8009 ± 0.0527	0.7399 ± 0.0625	0.8336 ± 0.0521	0.7653 ± 0.0572	0.8118 ± 0.0614	0.7903 ± 0.0572
	SMOTE	0.8209 ± 0.0568	0.8036 ± 0.0577	0.8403 ± 0.0597	0.7825 ± 0.0623	0.8232 ± 0.0553	0.8141 ± 0.0584
	Unaugmented	0.7707 ± 0.0551	0.7494 ± 0.062	0.8165 ± 0.0561	0.7858 ± 0.047	0.7863 ± 0.0509	0.7817 ± 0.0542
5000	Mod. Gamma-Poisson	0.838 ± 0.0521	0.8154 ± 0.0505	0.8353 ± 0.0618	0.7972 ± 0.0661	0.841 ± 0.055	0.8254 ± 0.0571
	Inter-Class	0.761 ± 0.0529	0.7852 ± 0.0543	0.7574 ± 0.0516	0.7473 ± 0.0553	0.7615 ± 0.0557	0.7625 ± 0.054
	Intra-Class	0.7661 ± 0.0505	0.7474 ± 0.0514	0.7785 ± 0.057	0.7006 ± 0.0663	0.7672 ± 0.04	0.752 ± 0.053
	Mod. Poisson	0.8221 ± 0.054	0.7969 ± 0.0548	0.8381 ± 0.046	0.7742 ± 0.0508	0.6887 ± 0.1054	0.784 ± 0.0622
	Replacement	0.801 ± 0.0569	0.7407 ± 0.063	0.8273 ± 0.0506	0.7688 ± 0.0631	0.8119 ± 0.0622	0.7899 ± 0.0591
	SMOTE	0.8157 ± 0.0562	0.7994 ± 0.0571	0.835 ± 0.0556	0.7693 ± 0.0581	0.823 ± 0.0567	0.8085 ± 0.0568
	Unaugmented	0.7707 ± 0.0551	0.7494 ± 0.062	0.8165 ± 0.0561	0.7858 ± 0.047	0.7863 ± 0.0509	0.7817 ± 0.0542

Table 19: Average balanced accuracy and standard deviation on out-of-domain CPTAC test set from 5x5 cross-validation with different classifier models and an overall average of these models for different augmentation methods. A portion of the training dataset (10%) was used for augmentation.

Class Size	Sampling Methods	EBM	KNN	Logistic	RF	SVM-RBF	Average
Max	Mod. Gamma-Poisson	0.6505 ± 0.054	0.5856 ± 0.0545	0.6016 ± 0.0734	0.6505 ± 0.0449	0.6129 ± 0.0682	0.6202 ± 0.059
	Inter-Class	0.6682 ± 0.0481	0.5847 ± 0.0645	0.63 ± 0.0743	0.6429 ± 0.0539	0.6321 ± 0.0751	0.6316 ± 0.0632
	Intra-Class	0.6587 ± 0.0446	0.6145 ± 0.0642	0.6173 ± 0.0633	0.6488 ± 0.0548	0.6249 ± 0.0674	0.6328 ± 0.0589
	Mod. Poisson	0.6467 ± 0.0493	0.6182 ± 0.0381	0.628 ± 0.0601	0.6574 ± 0.0718	0.6217 ± 0.0631	0.6344 ± 0.0565
	Replacement	0.6413 ± 0.0487	0.5949 ± 0.062	0.6199 ± 0.0731	0.6434 ± 0.0512	0.6135 ± 0.0766	0.6226 ± 0.0623
	SMOTE	0.6594 ± 0.0465	0.6013 ± 0.0501	0.6348 ± 0.0659	0.6649 ± 0.0563	0.6265 ± 0.0655	0.6374 ± 0.0569
	Unaugmented	0.6278 ± 0.052	0.5464 ± 0.0756	0.5707 ± 0.0739	0.5923 ± 0.0743	0.5848 ± 0.0915	0.5844 ± 0.0735
500	Mod. Gamma-Poisson	0.6904 ± 0.0564	0.6397 ± 0.0521	0.6184 ± 0.0775	0.6454 ± 0.0456	0.6348 ± 0.0539	0.6457 ± 0.0571
	Inter-Class	0.6638 ± 0.0461	0.6768 ± 0.0447	0.6358 ± 0.0653	0.6521 ± 0.0441	0.6492 ± 0.0456	0.6555 ± 0.0492
	Intra-Class	0.6408 ± 0.0673	0.5877 ± 0.076	0.5787 ± 0.0824	0.6239 ± 0.0581	0.5675 ± 0.079	0.5997 ± 0.0726
	Mod. Poisson	0.6329 ± 0.0576	0.6511 ± 0.0434	0.645 ± 0.0627	0.649 ± 0.0677	0.5279 ± 0.1103	0.6212 ± 0.0683
	Replacement	0.6469 ± 0.0424	0.5822 ± 0.0534	0.6169 ± 0.0765	0.6488 ± 0.0583	0.6184 ± 0.0699	0.6226 ± 0.0601
	SMOTE	0.6403 ± 0.0496	0.6155 ± 0.0366	0.6291 ± 0.0642	0.6294 ± 0.0679	0.6271 ± 0.0567	0.6283 ± 0.055
	Unaugmented	0.6278 ± 0.052	0.5464 ± 0.0756	0.5707 ± 0.0739	0.5923 ± 0.0743	0.5848 ± 0.0915	0.5844 ± 0.0735
5000	Mod. Gamma-Poisson	0.6973 ± 0.0389	0.6553 ± 0.056	0.629 ± 0.0587	0.6574 ± 0.0521	0.634 ± 0.052	0.6546 ± 0.0515
	Inter-Class	0.6233 ± 0.0531	0.6551 ± 0.0326	0.6189 ± 0.0723	0.6277 ± 0.0386	0.6382 ± 0.0426	0.6327 ± 0.0479
	Intra-Class	0.6256 ± 0.0492	0.5586 ± 0.0534	0.5556 ± 0.0965	0.5921 ± 0.0575	0.5619 ± 0.0777	0.5788 ± 0.0669
	Mod. Poisson	0.63 ± 0.0531	0.6494 ± 0.0411	0.6314 ± 0.0601	0.6465 ± 0.0469	0.5286 ± 0.1039	0.6172 ± 0.061
	Replacement	0.6448 ± 0.0453	0.5808 ± 0.0547	0.6131 ± 0.0805	0.64 ± 0.0704	0.6165 ± 0.0703	0.619 ± 0.0642
	SMOTE	0.6454 ± 0.0483	0.6223 ± 0.0366	0.6243 ± 0.0681	0.6165 ± 0.0732	0.6253 ± 0.0599	0.6268 ± 0.0572
	Unaugmented	0.6278 ± 0.052	0.5464 ± 0.0756	0.5707 ± 0.0739	0.5923 ± 0.0743	0.5848 ± 0.0915	0.5844 ± 0.0735

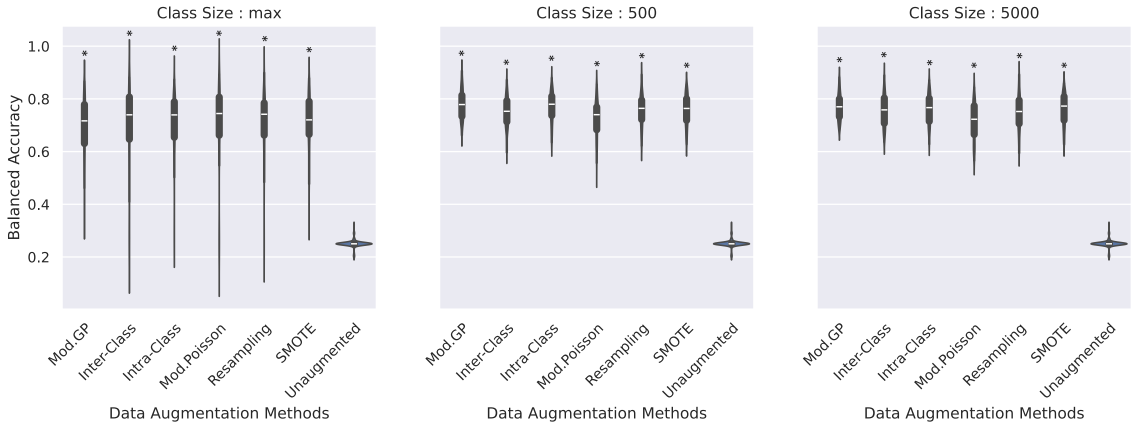

Figure 16: Classification performance on generated samples from a VAE pre-trained on augmented data, sampled from a distribution modelled on in-domain TCGA test set. Although the methods show a similar performance, on average, the Mod. GP augmentation method is associated with the best score($\mathbf{77.71\% \pm 0.0536}$). The VAEs generating the samples are trained on 10% data augmented by the different methods as shown in the figure. $*$ indicates the method is significantly better than the unaugmented case.

Table 20: Average balanced accuracy and standard deviation of different classifier models on generated samples from a VAE pre-trained on augmented data, sampled from a distribution modelled on in-domain TCGA test set from 5x5 cross-validation and an overall average of these models for different augmentation methods. A portion of the real training dataset (10%) was used for augmentation and each class was augmented to size 500.

Sampling Methods	EBM	KNN	Logistic	RF	SVM-RBF	Average
Mod. Gamma-Poisson	0.7794 ± 0.0548	0.7694 ± 0.0504	0.7829 ± 0.0565	0.7689 ± 0.0553	0.7851 ± 0.0508	0.7771 ± 0.0536
Inter-Class	0.749 ± 0.0558	0.7362 ± 0.058	0.7609 ± 0.0515	0.7524 ± 0.0538	0.7619 ± 0.0465	0.7521 ± 0.0531
Intra-Class	0.76 ± 0.0524	0.7568 ± 0.0526	0.7877 ± 0.055	0.7677 ± 0.0561	0.7763 ± 0.0578	0.7697 ± 0.0548
Mod. Poisson	0.7322 ± 0.0714	0.7314 ± 0.0611	0.7327 ± 0.0626	0.7237 ± 0.0839	0.7255 ± 0.0681	0.7291 ± 0.0694
Replacement	0.7612 ± 0.0516	0.7396 ± 0.0687	0.7719 ± 0.0565	0.7567 ± 0.0561	0.7623 ± 0.0573	0.7583 ± 0.0580
SMOTE	0.7639 ± 0.0629	0.7468 ± 0.0528	0.7619 ± 0.0599	0.7544 ± 0.0569	0.7585 ± 0.0548	0.7571 ± 0.0575
Unaugmented	0.2517 ± 0.0091	0.2502 ± 0.0122	0.2514 ± 0.0209	0.2504 ± 0.0162	0.25 ± 0.0	0.2507 ± 0.0117

Table 21: Average balanced accuracy and standard deviation of different classifier models on generated samples from a VAE pre-trained on augmented data, sampled from a distribution modelled on out-of-domain CPTAC test set from 5x5 cross-validation and an overall average of these models for different augmentation methods. A portion of the real training dataset (10%) was used for augmentation and each class was augmented to size 500.

Sampling Methods	EBM	KNN	Logistic	RF	SVM-RBF	Average
Mod. Gamma-Poisson	0.6223 ± 0.0574	0.6018 ± 0.0785	0.6009 ± 0.059	0.5978 ± 0.0653	0.6157 ± 0.0704	0.6077 ± 0.0661
Inter-Class	0.649 ± 0.0632	0.6719 ± 0.0657	0.67 ± 0.0622	0.6489 ± 0.0602	0.678 ± 0.0635	0.6636 ± 0.0630
Intra-Class	0.6373 ± 0.0754	0.638 ± 0.0585	0.6502 ± 0.0624	0.6225 ± 0.0675	0.6448 ± 0.064	0.6386 ± 0.0656
Mod. Poisson	0.6147 ± 0.066	0.6201 ± 0.0657	0.6305 ± 0.0628	0.5892 ± 0.0621	0.6164 ± 0.0569	0.6142 ± 0.0627
Replacement	0.6198 ± 0.0653	0.5848 ± 0.0708	0.6254 ± 0.0525	0.6014 ± 0.0739	0.6147 ± 0.0568	0.6092 ± 0.0649
SMOTE	0.6203 ± 0.0546	0.617 ± 0.0631	0.624 ± 0.0528	0.6094 ± 0.0616	0.6209 ± 0.0583	0.6183 ± 0.0581
Unaugmented	0.2573 ± 0.0153	0.2512 ± 0.0069	0.2524 ± 0.0213	0.2536 ± 0.0162	0.25 ± 0.0	0.2529 ± 0.0119

Table 22: Average balanced accuracy and standard deviation on in-domain TCGA test data of different classifier models in predicting the MSI status from embeddings retrieved from a VAE and an overall average of these models for different augmentation methods. A portion of the real training dataset (10%) was used for augmentation and each class was augmented to size 500.

Sampling Methods	EBM	KNN	Logistic	RF	SVM-RBF	Average
Mod. Gamma-Poisson	0.5723 ± 0.0483	0.5958 ± 0.0472	0.6117 ± 0.0597	0.5895 ± 0.0617	0.5865 ± 0.0713	0.5912 ± 0.0576
Inter-Class	0.5683 ± 0.0423	0.6184 ± 0.0498	0.6216 ± 0.0667	0.5962 ± 0.0643	0.6187 ± 0.0578	0.6046 ± 0.0562
Intra-Class	0.5667 ± 0.0538	0.6177 ± 0.0534	0.6222 ± 0.0691	0.5983 ± 0.0587	0.6223 ± 0.062	0.6054 ± 0.0594
Mod. Poisson	0.5736 ± 0.0601	0.5879 ± 0.0677	0.6023 ± 0.0615	0.5887 ± 0.0472	0.5772 ± 0.0555	0.5859 ± 0.0584
Replacement	0.6057 ± 0.0572	0.5872 ± 0.0543	0.6391 ± 0.0482	0.619 ± 0.0467	0.6124 ± 0.06	0.6127 ± 0.0533
SMOTE	0.5875 ± 0.0542	0.5707 ± 0.0548	0.6229 ± 0.0598	0.6149 ± 0.0591	0.5945 ± 0.062	0.5981 ± 0.0580
Unaugmented	0.4873 ± 0.0385	0.483 ± 0.0349	0.484 ± 0.04	0.4862 ± 0.0406	0.4932 ± 0.0191	0.4867 ± 0.0346

Table 23: Average balanced accuracy and standard deviation on out-of-domain CPTAC test data of different classifier models in predicting the MSI status from embeddings retrieved from a VAE and an overall average of these models for different augmentation methods. A portion of the real training dataset (10%) was used for augmentation and each class was augmented to size 500.

Sampling Methods	EBM	KNN	Logistic	RF	SVM-RBF	Average
Mod. Gamma-Poisson	0.5808 ± 0.0826	0.6444 ± 0.0957	0.6823 ± 0.1153	0.6208 ± 0.0896	0.6458 ± 0.1118	0.6348 ± 0.0990
Inter-Class	0.6601 ± 0.1057	0.7332 ± 0.1307	0.7087 ± 0.1329	0.6897 ± 0.104	0.728 ± 0.1256	0.7039 ± 0.1198
Intra-Class	0.6394 ± 0.0859	0.7097 ± 0.1083	0.7333 ± 0.122	0.7016 ± 0.1163	0.7592 ± 0.1111	0.7086 ± 0.1087
Mod. Poisson	0.6345 ± 0.0792	0.6534 ± 0.1046	0.6996 ± 0.107	0.668 ± 0.0658	0.6463 ± 0.097	0.6604 ± 0.0907
Replacement	0.6495 ± 0.0701	0.637 ± 0.1028	0.7616 ± 0.1182	0.7021 ± 0.1014	0.6915 ± 0.1133	0.6883 ± 0.1012
SMOTE	0.6152 ± 0.0916	0.5956 ± 0.1044	0.6992 ± 0.1062	0.6892 ± 0.0947	0.6456 ± 0.089	0.6489 ± 0.0972
Unaugmented	0.5002 ± 0.0274	0.5144 ± 0.0411	0.4903 ± 0.0425	0.5078 ± 0.0374	0.4989 ± 0.0265	0.5023 ± 0.0350

Table 24: Average balanced accuracy and standard deviation on in-domain TCGA test data of different classifier models in predicting the CIMP status from embeddings retrieved from a VAE and an overall average of these models for different augmentation methods. A portion of the real training dataset (10%) was used for augmentation and each class was augmented to size 500.

Sampling Methods	EBM	KNN	Logistic	RF	SVM-RBF	Average
Mod. Gamma-Poisson	0.4329 ± 0.0757	0.5189 ± 0.0562	0.5275 ± 0.0344	0.5153 ± 0.0572	0.4867 ± 0.062	0.4963 ± 0.0571
Inter-Class	0.4606 ± 0.0789	0.5633 ± 0.0572	0.5565 ± 0.0489	0.5183 ± 0.0597	0.5156 ± 0.0671	0.5229 ± 0.0624
Intra-Class	0.4538 ± 0.078	0.5358 ± 0.0571	0.5451 ± 0.0476	0.5085 ± 0.0688	0.5076 ± 0.0624	0.5102 ± 0.0628
Mod. Poisson	0.4418 ± 0.0718	0.5024 ± 0.0705	0.5302 ± 0.0538	0.531 ± 0.0763	0.4818 ± 0.0797	0.4974 ± 0.0704
Replacement	0.4502 ± 0.0639	0.514 ± 0.0661	0.542 ± 0.0526	0.5288 ± 0.0801	0.5127 ± 0.0645	0.5095 ± 0.0654
SMOTE	0.4595 ± 0.0902	0.4968 ± 0.0615	0.5441 ± 0.0559	0.5308 ± 0.0699	0.5018 ± 0.0779	0.5066 ± 0.0711
Unaugmented	0.3452 ± 0.0392	0.3429 ± 0.0595	0.3363 ± 0.032	0.3411 ± 0.0646	0.3386 ± 0.0183	0.3408 ± 0.0427

References

A. F. Agarap. Deep learning using rectified linear units (relu). *arXiv preprint arXiv:1803.08375*, 2018.

T. Barry. Tim barry: Gamma, poisson, and negative binomial distributions, 2020. URL `https://timothy-barry.github.io/posts/2020-06-16-gamma-poisson-nb/`.

S. A. Buechler, M. T. Stephens, A. B. Hummon, K. Ludwig, E. Cannon, T. C. Carter, J. Resnick, Y. Gökmen-Polar, and S. S. Badve. Colotype: a forty gene signature for consensus molecular subtyping of colorectal cancer tumors using whole-genome assay or targeted rna-sequencing. *Scientific reports*, 10(1):1–13, 2020.

Synthetic Data Metrics. DataCebo, Inc., 02 2025. URL `https://docs.sdv.dev/sdmetrics/`. Version 0.19.0.

M. Greenwood and G. U. Yule. An inquiry into the nature of frequency distributions representative of multiple happenings with particular reference to the occurrence of multiple attacks of disease or of repeated accidents. *Journal of the Royal statistical society*, 83(2):255–279, 1920.

T. Heide, J. Househam, G. D. Cresswell, I. Spiteri, C. Lynn, M. Mossner, C. Kimberley, J. Fernandez-Mateos, B. Chen, L. Zapata, et al. The co-evolution of the genome and epigenome in colorectal cancer. *Nature*, 611(7937):733–743, 2022.

J. Househam, T. Heide, G. D. Cresswell, I. Spiteri, C. Kimberley, L. Zapata, C. Lynn, C. James, M. Mossner, J. Fernandez-Mateos, et al. Phenotypic plasticity and genetic control in colorectal cancer evolution. *Nature*, 611(7937):744–753, 2022.

D. P. Kingma and J. Ba. Adam: A method for stochastic optimization. *arXiv preprint arXiv:1412.6980*, 2014.

L. McInnes, J. Healy, and J. Melville. Umap: Uniform manifold approximation and projection for dimension reduction. *arXiv preprint arXiv:1802.03426*, 2018.

F. Pedregosa, G. Varoquaux, A. Gramfort, V. Michel, B. Thirion, O. Grisel, M. Blondel, P. Prettenhofer, R. Weiss, V. Dubourg, J. Vanderplas, A. Passos, D. Cournapeau, M. Brucher, M. Perrot, and E. Duchesnay. Scikit-learn: Machine learning in Python. *Journal of Machine Learning Research*, 12:2825–2830, 2011.
